# *Amalaki rasayana*, a traditional Indian drug enhances cardiac mitochondrial and contractile functions and improves cardiac function in rats with hypertrophy

**DOI:** 10.1038/s41598-017-09225-x

**Published:** 2017-08-17

**Authors:** Vikas Kumar, kumar A. Aneesh, K. Kshemada, Kumar G. S. Ajith, Raj S. S. Binil, Neha Deora, G. Sanjay, A. Jaleel, T. S. Muraleedharan, E. M. Anandan, R. S Mony, M. S. Valiathan, Kumar T. R. Santhosh, C. C Kartha

**Affiliations:** 1 Cardiovascular Diseases and Diabetes Biology, Rajiv Gandhi Center for Biotechnology (RGCB), Trivandrum, India; 2 Department of Cardiology, Sree Chitra Tirunal Institute for Medical Sciences & Technology, Trivandrum, India; 3 Centre for Bio-Separation Technology, Vellore Institute of Technology, Vellore, India; 4 Kottakal Arya Vaidyasala, Kottakkal, Kerala India; 5 Manipal University, Manipal, Karnataka India

## Abstract

We evaluated the cardioprotective effect of Amalaki Rasayana (AR), a rejuvenating Ayurvedic drug prepared from *Phyllanthus* *emblica* fruits in the reversal of remodeling changes in pressure overload left ventricular cardiac hypertrophy (LVH) and age-associated cardiac dysfunction in male Wistar rats. Six groups (aging groups) of 3 months old animals were given either AR or ghee and honey (GH) orally; seventh group was untreated. Ascending aorta was constricted using titanium clips in 3 months old rats (N = 24; AC groups) and after 6 months, AR or GH was given for further 12 months to two groups; one group was untreated. Histology, gene and protein expression analysis were done in heart tissues. Chemical composition of AR was analyzed by HPLC, HPTLC and LC-MS. AR intake improved (*P* < 0.05) cardiac function in aging rats and decreased LVH (*P* < 0.05) in AC rats as well as increased (*P* < 0.05) fatigue time in treadmill exercise in both groups. In heart tissues of AR administered rats of both the groups, SERCA2, CaM, Myh11, antioxidant, autophagy, oxidative phosphorylation and TCA cycle proteins were up regulated. ADRB1/2 and pCREB expression were increased; pAMPK, NF-kB were decreased. AR has thus a beneficial effect on myocardial energetics, muscle contractile function and exercise tolerance capacity.

## Introduction

Ayurveda which signifies the science of long life, is one of the ancient (>5000 years old) systems of medicine in India. Health promotion, disease prevention and rejuvenation approaches are used in this system of medicine through dietary and therapeutic means and both approaches can slow aging and invigorate functions of the body’s organs^1–3^. Rejuvenation and remodeling strategies comprise the ‘Rasayana chikitsa’ (rejuvenation therapy) in Ayurveda. Rasayana (‘Rasa’: plasma; Ayana: path; which means the path that ‘Rasa’ takes) group is a class of medicinal plants in Ayurvedic pharmacology used for this purpose. ‘Rasayana’ drugs refurbish the neuronal, endocrinal and immune systems and are considered to prevent ageing, re-establish youth, strengthen life, brain power and prevent diseases, thus enhancing bodily resistance to all kinds of injury^2, 3^.

Amalaki rasayana/AR (obtained from the fruits of *Phyllanthus* *emblica* *or Embilica* *officinalis*; Family: Euphorbiaceae, Aurvedic name: ‘Amala’) is an Indian traditional Ayurvedic drug used as a rejuvenating medicine in aging conditions. The fruits of Amala commonly used in Aryuveda are assumed to enhance defense against diseases. Ayurveda literature mentions Amalaki to have a beneficial role in cancer, diabetes, liver and heart diseases, gastric ulcers and various other disorders. Amalaki rasayana has antioxidant, immunomodulatory, antipyretic, analgesic, cytoprotective, antitussive and gastroprotective actions. It is also used for memory enhancement and for lowering blood cholesterol levels. Reports from various scientific studies suggest that its consumption also protects tissues against radiation damage^4–7^. In recent studies, AR was seen to increase the median life span and starvation resistance in Drosophila melanogaster^8^ and increase the levels of apoptosis inhibitory proteins (DIAPs) and decrease caspases and levels of Rpr, Hid or Grim (RHG) pro-apoptotic proteins in the eye disc and salivary glands of AR treated Drosophila^9^. AR fed aged rats were also found to have increased genome stability in astrocytes and neurons of the cerebral cortex^10^.

In recent times, there is mounting interest in the use of natural products as secondary medicines for cardiovascular disease. Juan Guo *et al*. in 2011, demonstrated that alcoholic ginseng extract can inhibit cardiomyocyte hypertrophy and heart failure through Na^+^-H^+^ exchanger-1(NHE-1) and inhibit and attenuate calcineurin activation^11^. Bhattacharya SK *et al*. studied the effect of tannoid principles of fresh juice of emblica fruits on ischemia-reperfusion (IRI)-induced oxidative stress in the rat heart. Administration of tannoid principle prevents IRI-induced effect, when given orally twice daily for 14 days^12^. Rajak S *et al*. discovered that chronic administration of Amala improved antioxidant defense of myocardium in IRI induced through oxidative stress. Their results indicate that long term administration of fresh Amala fruit homogenate (500 and 750 mg/kg) can augment endogenous antioxidants and protect rat hearts from associated oxidative stress in IRI^13^. Bhatia J *et al*. evaluated the effect of hydroalcoholic lyophilized extract of emblica in rats with hypertension induced by deoxycorticosterone acetate or 1% sodium chloride high salt (DOCA/HS) administration^14^. They reported that *Emblica* *officinalis* reduces oxidative stress and prevents development and progression of hypertension by modulating levels of serum NO, activated eNOS endogenous antioxidants, and electrolytes. Studies by Yokozawa *et al*. indicate that Amala may attenuates oxidative stress and may prevent hyperlipidemia associated with aging^15^.

These studies prompted us to evaluate the effect of AR in the reversal of remodeling changes in pressure overload left ventricular cardiac hypertrophy and age-associated cardiac dysfunction in rats. No *in vivo* studies have been previously done to evaluate the cardio protective effect of AR in pressure overload hypertrophy and cardiac function in aging animals.

We observed that long term oral intake of AR improves cardiac function in aged rats as well as in rats with pressure overload left ventricular hypertrophy. The functional improvement was associated with enhanced myocardial contractile function and mitochondrial bioenergetics.

## Results

### Characterization of AR (Amalaki rasayana)

Results of qualitative solubility analysis of AR and a mixture of ghee and honey (GH) used as carrier in AR in different solvents are given in Supplementary Table S1A,B,C. RP-HPLC (Reverse phase–high performance liquid chromatography) profile of AR dissolved in ethanol was different when compared with GH in the same solvent (Supplementary Fig. S1b,d; Supplementary Table S1A,C). AR had good solubility in acetonitrile and ethanol and moderate solubility in methanol. We observed different peaks at retention time of 0.897, 2.030, 4.900, 8.109 and 9.465 min from the RP-HPLC profile of AR (Supplementary Fig. S1b,d; Supplementary Table S1C). The RP-HPLC profile of AR in acetonitrile also showed different peaks at retention time of 2.155, 2.568, 5.482, 7.985, 18.026 and 18.210 min when compared with GH (Supplementary Fig. S1c,e; Supplementary Table S1C).

HPTLC profiles of samples of the finished formulation revealed the presence of gallic acid and ellagic acid (Supplementary Fig. S1a and Supplementary Table S2A,B).

LC-MS analysis of lyophilized powder of AR revealed enrichment of components such as putative anti-inflammatory arachidonate (eicosatetraenoic acid), norepinephrine sulfate and vitamin metabolites, as identified from online software XCMS for metabolomics study (Supplementary Fig. S1f; Table 1).

**Table 1:** List of components from AR, tentatively identified after LC-MS method by XCMS analysis.

ID	Compound name	m/z ratio	Match form	Intensity (Maximum)	RT (Min)	Associated pathway
C00169	Carbamoyl phosphate	141.9899	M + H[1+]	2.09E + 03	18.29	Pyrimidine metabolism
C00073	L-Methionine; Methionine; L-2-Amino-4methylthiobutyric acid	150.0575	M + H[1+]	2.93E + 03	15.54	Amino acid metabolism
C00647	Pyridoxamine phosphate	249.0617	M + H[1+]	1.22E + 03	8.72	Vitamin B6 (pyridoxine) metabolism
**nrpphrsf**	**Sulfate derivative of norepinephrine**	**250**.**0401**	**M** + **H**[**1**+]	**7**.**82E** + **02**	**14**.**72**	**Tyrosine metabolism**
C05841	Nicotinate D-ribonucleoside	279.0692	M + Na[1+]	1.71E + 03	0.79	Vitamin B3 (nicotinate and nicotinamide) metabolism
**CE2961**	**4**-**hydroxy**-**all**-**trans**-**retinyl acetate**	**328**.**218**	**M**-**NH**_**3**_ + **H**[**1**+]	**6**.**74E** + **02**	**6**.**31**	**Vitamin A** (**retinol**) **metabolism**
**CE2567**	**5**(**S**),**6**(**S**)-**epoxy**-**15**(**S**)-**hydroxy**-**7E**,**9E**,**11Z**,**13E**-**eicosatetraenoic acid**	**334**.**2144**	**M** + **H**[**1**+]	**2**.**40E** + **03**	**13**.**64**	**Arachidonic acid metabolism or putative anti inflammatory metabolite**
C00959	Prostaglandin B1	359.2163	M + Na[1+]	1.83E + 03	15.44	Prostaglandin formation from arachidonate
C05552	N6-D-Biotinyl-L-lysine; Biocytin; epsilon-N-Biotinyl-L-lysine	373.192	M + H[1+]	5.38E + 03	14.3	Vitamin H (biotin) metabolism
CE5139	12-oxo-20-dihydroxy-leukotriene B4	388.1884	M + Na[1+]	1.09E + 04	14.33	Leukotriene metabolism
C00144	Guanosine 5’-monophosphate; Guanylic acid	402.0196	M + K[1+]	1.04E + 03	7.98	Purine metabolism
C00695	12alpha-Trihydroxy-5beta-cholanic acid; Cholic acid	409.2919	M + H[1+]	4.96E + 03	15.51	Bile acid metabolism
CE7145	13’-carboxy-alpha-tocotrienol	454.3103	M + H[1+]	1.54E + 04	15.63	Vitamin E metabolism
**CE2205**	**1alpha**,**24R**,**25**-**trihydroxyvitamin D3**	**455**.**3133**	**M** + **Na**[**1**+]	**4**.**43E** + **03**	**15**.**63**	**Vitamin D3** (**cholecalciferol**) **metabolism**
C00664	5-Formiminotetrahydrofolate	473.1939	M + H[1+]	9.78E + 02	13.61	Vitamin B9 (folate) metabolism, Histidine metabolism
C06453	Methylcobalamin	1382.5472	M + K[1+]	3.93E + 03	0.68	Vitamin B12 (cyanocobalamin) metabolism

### Cytotoxicity assay

AR did not change the morphological features of H9c2 cells. AR was not found to be cytotoxic at different doses (maximum dose of 100 mg/ml) for up to 72 hours (Supplementary Fig. S1g).

### Animal experiments

#### Aging group

Significant differences in ECG parameters such as P wave, QRS interval, R-R interval (seconds), QT interval were not observed in any of the animals. A mean blood pressure of 116–120 mmHg was noted in rats of all the groups. Heart rates were also within normal limits in all the rats.

**Figure 1:**
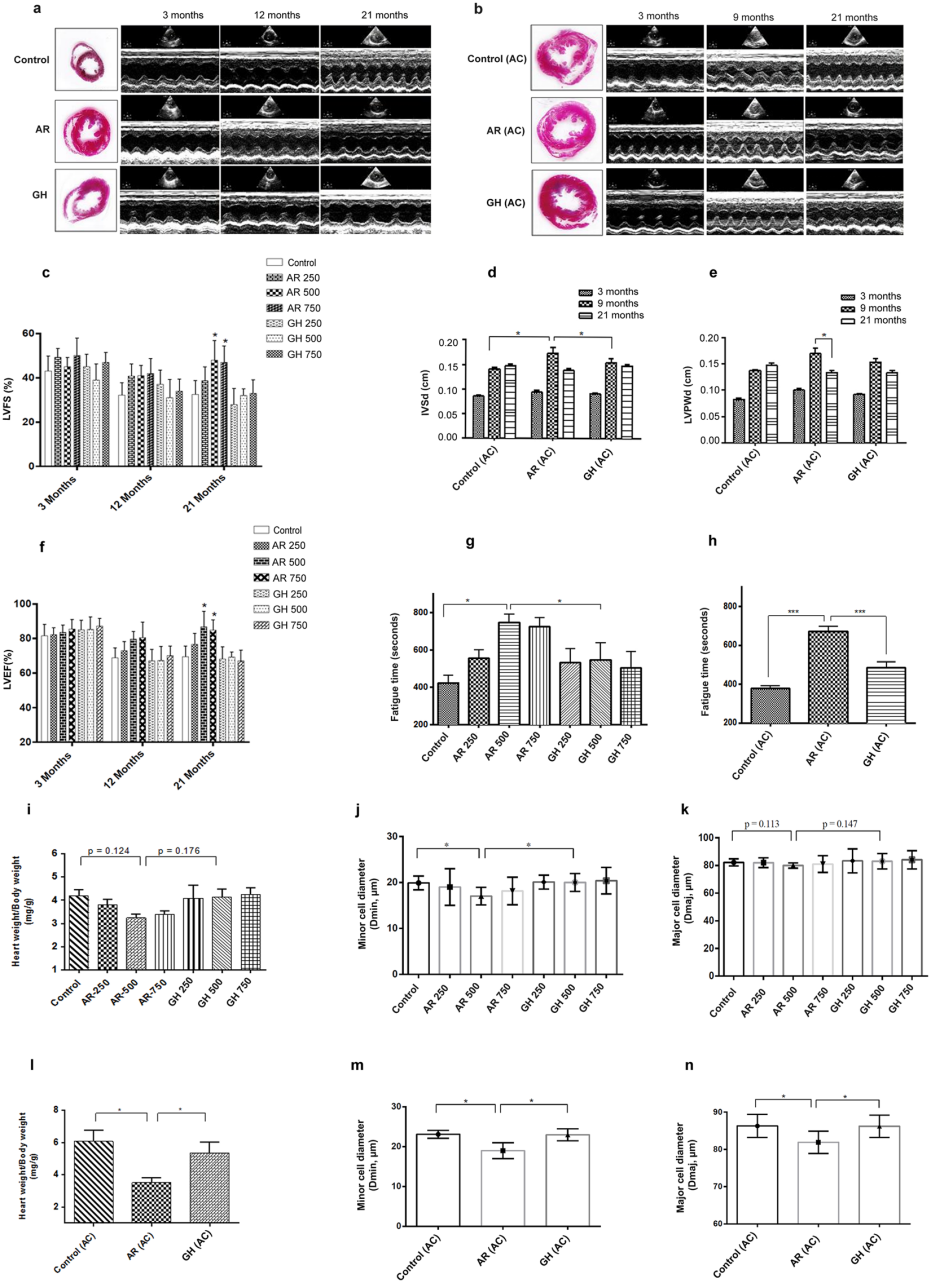
AR improved the left ventricular function in aging and aorta constricted group of rats. (**a**) Trichrome stained heart sections from 21 months old rats and representative M- mode echocardiography images of 3, 12 and 21 months old rats suggests unaltered left ventricular dimensions in AR treated rats. (**b**) Trichrome stained heart sections of 21 months rats with left ventricular hypertrophy (LVH) and representative M- mode echocardiography images of 3, 9 and 21 months rats with constricted aorta suggests sustained LV dimensions in AR treated rats. (**c**,**f**) Echocardiography measurements represents improved left ventricle dimensions and function in AR treated rats of aging group; LVEF (left ventricular ejection fraction), LVFS (left ventricular fractional shortening). (**d**,**e**) Echocardiography measurements suggests sustained left ventricle dimensions and function in AR administered rats of AC group. IVSd (diastolic interventricular septal thickness), LVPWd (diastolic left ventricle posterior wall thickness). (**g**) Fatigue times of 21 months old rats given different doses of AR or GH and control rats. (**h**) Fatigue times of 21 months old rats with constricted aorta and left ventricular hypertrophy. (**i**,**l**) Heart weight/body weight (mg/g) was lesser in AR administered rats and had reduced severe hypertrophy when compared with GH or control group in aging group. (**j**,**k**) Minor cell diameter (Dmin, μm) and major cell diameter (Dmaj, μm) were lesser and revealed lesser hypertrophy in AR administered rats of aging group. (**m**,**n**) Dmin and Dmaj were lesser (p < 0.05) and suggest lesser cardiomyocyte size in AR administered rats when compared with GH or control group in aorta constricted group. Response was better with higher doses (500 or 750 mg/kg, body weight) of AR when compared with AR-250. Data are represented as the mean ± s.e.m from 8 rats in each group. *p < 0.05 AR versus GH/control and ***P < 0.001 AR (AC) versus GH (AC) or control (AC) groups, results were analyzed by 2-way ANOVA followed by multiple comparisons with Tukey’s test.

In experiment 1, among the echo parameters, left ventricular fraction shortening (LVFS) and left ventricular ejection fraction (LVEF) were found to be significantly (*P* < 0.05) improved in AR administered group of rats, when compared to GH treated/control rats. The animals which received larger doses of AR had more beneficial effects than those which received 250 mg/kg body weight (Fig. 1a,c,f). Intraventricular septal thickness in diastole (IVSd), intraventricular septal thickness in systole (IVSs), left ventricular internal dimension in systole (LVIDs), left ventricular internal dimension in diastole (LVIDd), left ventricular posterior wall thickness in systole (LVPWs), left ventricular posterior wall thickness in diastole (LVPWd) were not different among different experimental groups (Supplementary Table S3).

AR improved exercise tolerance capacity of aged rats. Fatigue time in treadmill exercise was found to be significantly (*P* < 0.05) increased in AR administered rats, when compared with rats of other experimental groups. Fatigue times were more in the animals which received larger doses of AR than those which received 250 mg/kg body weight (Fig. 1g).

**Figure 2:**
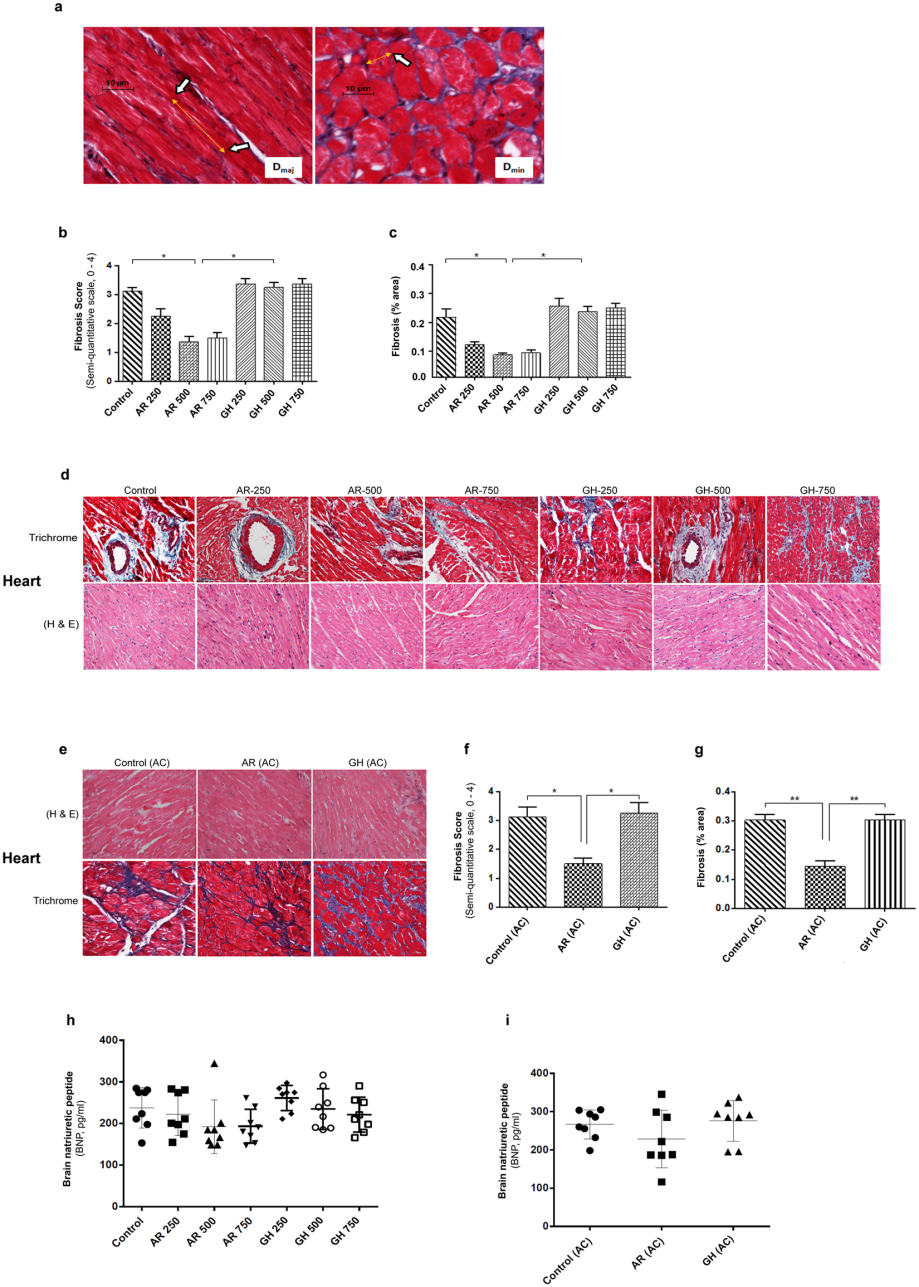
AR reduces cardiac fibrosis induced by aging and pressure overload. (**a**) Representative image of quantification of cardiomyocyte length (D_maj_) and diameter (D_min_) measured from trichrome stained heart sections from 21 months aged rats. (**b**,**c**,**d**) Fibrotic score and fibrosis area were decreased (response was better with higher doses) in AR administered rats in aging group. (**e**,**f**,**g**) Quantification of fibrosis revealed that rats of aorta constricted group have myocyte hypertrophy and interstitial fibrosis and AR administered rats have lesser myocardial fibrosis. (**d**,**e**) Representative images of Hematoxylin and Eosin and trichrome stained myocardium (20X) of aging and aorta constricted group. (**h**)BNP levels in serum in 21 months old rats in aging groups administered different doses of AR or GH and control rats. (**i**) BNP levels in serum of 21 months old rats in aorta constricted groups. Values represents the mean ± s.e.m. of data from 8 rats in each group. *P < 0.05 (AR versus GH/control groups), **P < 0.01 (AR (AC) versus GH (AC)/control (AC) groups), results were analyzed by 2-way ANOVA followed by multiple comparisons with Tukey’s test.

Heart weight/body weight (HW/BW; mg/g) ratios in AR, GH administered and control rats are given in Supplementary Table S6. The ratio was less in AR administered rats though statistically significant difference was not seen compared to GH and control groups (Fig. 1i). Major cell diameter (Dmaj; μm) and Minor cell diameter (Dmin; μm) (equivalent to cardiomyocyte length) of cardiomyocytes were found to be decreased in AR administered rats (Figs 1j,k and 2a, Supplementary Table S5). Fibrosis score and fibrosis (% area) analysis from trichrome stained heart sections also revealed mild interstitial fibrosis in AR treated rats while all the rats of GH treated or control rats had moderate degree of myocardial fibrosis (Fig. 2b,c,d). Rats which received larger doses of AR had less fibrosis than those which received 250 mg/kg body weight. BNP levels in AR administered group of rats when compared with other groups were not significantly different irrespective of the dose of AR. However, a mean BNP level >150 pg/ml was observed in all the groups of rats (Fig. 2h).

To observe toxicity or other adverse changes after AR administration, we also recorded absolute and relative weights of lungs, livers and kidneys and did histopathology of tissues from these organs of rats after sacrificing at the age of 21 months. Absolute tissue weight, tissue weight/body weight ratio and histology did not reveal significant weight gain/loss, denuding bronchiolitis or perivascular cuffing in the lungs, steatosis or hepatiits in liver or glomerular sclerosis or tubular lesions in the kidney (Supplementary Table S6).

Echocardiography and exercise tolerance studies as well as histology revealed that 500 and 750 mg/kg body weight of AR had similar effects. The response to these dosages was significantly better compared to those to 250 mg/kg body weight of AR. Hence we chose tissues of rats administered with 500 mg/kg body weight for molecular analysis. The same dose was also selected for administration in rats with LVH in the AC group.

#### Aorta constriction group

Significant differences were not observed in the ECG parameters in any of the rats in this group. Mean blood pressure (BP) was 125–130 mmHg in rats of all the groups. Mean heart rates (HR) were also within normal limits in all the animals.

Echocardiography confirmed left ventricular hypertrophy in all the rats, 6 months after constriction of ascending aorta (Fig. 1b,d,e). There was significant increase in IVSd, LVPWd (*P* < 0.001) and significant decrease in LVIDd, LVIDs (*P* < 0.001) at the end of 6 months (Supplementary Table S4). AR treatment significantly (*P* < 0.05) decreased the IVSd and LVPWd at the end of 21 months (12 months after treatment with AR) in rats with left ventricular hypertrophy when compared with GH administered rats with left ventricular hypertrophy (Fig. 1b,d,e, Supplementary Table S4). There were no differences in LVEF and LVFS in AR administered rats when compared with other groups.

AR administration increased the fatigue time in rats with constriction of aorta and administered AR. Exercise tolerance capacity was also found to be significantly (*P* < 0.001) increased in the rats with left ventricular hypertrophy and were administered AR (Fig. 1h).

HW/BW ratios (mg/g) were significantly (*P* < 0.05) different among AR, GH administered or control rats (Fig. 1l). Dmaj and Dmin of cardiomyocytes were found to be decreased in AR administered rats (Fig. 1m,n and Supplementary Table S5). Both HW/BW ratio and cardiomyocyte dimensions assessment revealed lesser myocyte hypertrophy in AR administered rats of AC group when compared with control or GH group.

Fibrosis score and fibrosis (% area) analysis from trichrome stained heart sections also revealed mild interstitial fibrosis in AR treated rats while all the rats of GH treated or control rats had moderate degree of myocardial fibrosis (Fig. 2e,f,g).

We did not find significant differences in BNP levels in AR administered group of rats when compared with other groups. Mean BNP levels were however, >200 pg/ml at the end of 18 months after constriction in rats which underwent constriction of aorta (Fig. 2i).

### Gene and protein expression analysis

**Figure 3:**
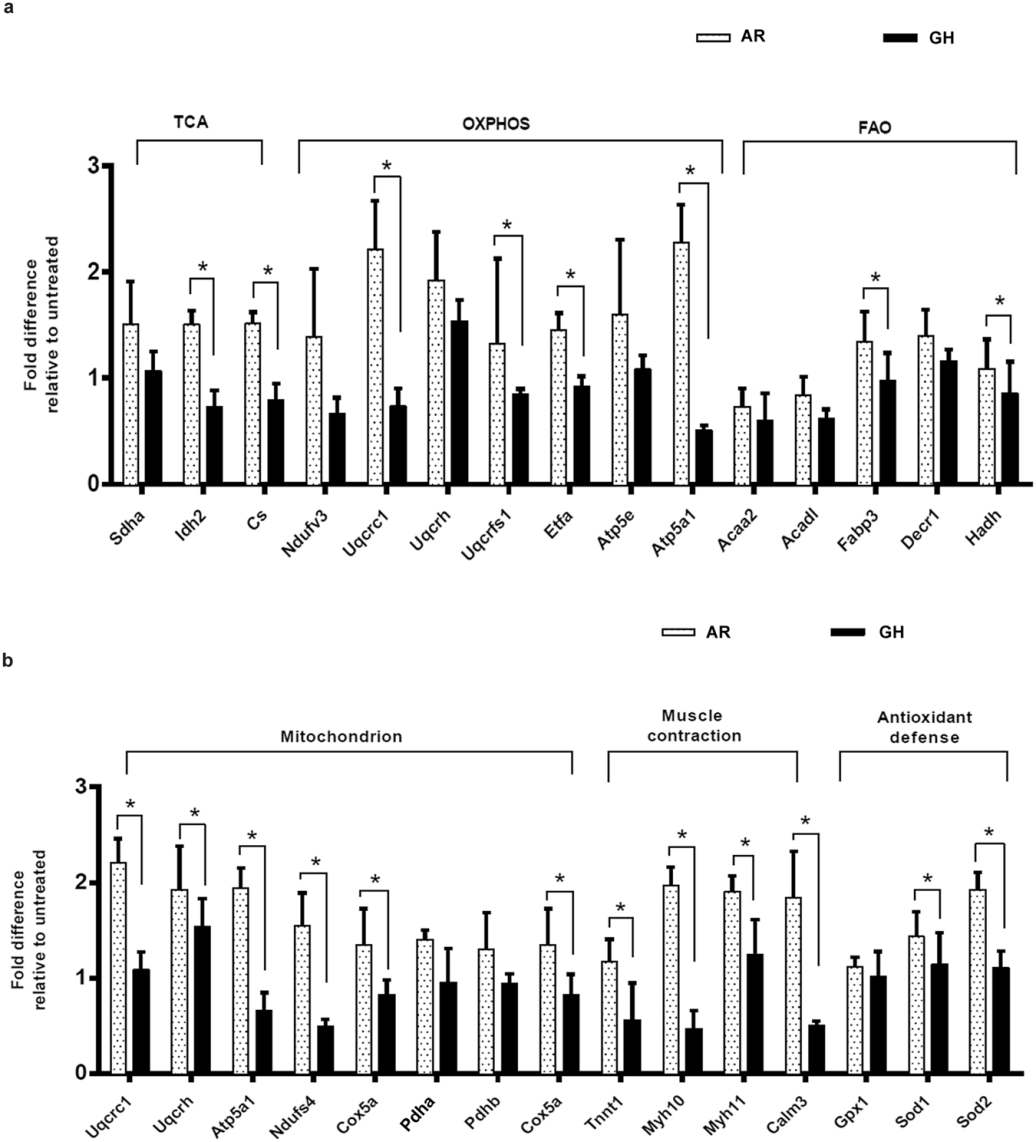
AR improved whole cardiometabolic profile suggests increased bioenergetics function in myocardium of aged rats. (**a**) LC-MS analysis suggests increased expression of proteins regulating the mitochondrial tricarboxylic acid cycle, oxidative phosphorylation (OXPHOS) and fatty acid oxidation pathways in heart tissues of AR administered rats. (**b**) A significant fold differences are seen in the expression of proteins regulating the mitochondrial OXPHOS, muscle contraction and antioxidant defense pathways. Sdha: Succinate dehydrogenase a, Idh2: Isocitrate dehydrogenase 2, Cs: Citrate synthase, Ndufv3: NADH dehydrogenase ubiquinone flavoprotein 3, Uqcrc1: Ubiquinone cytochrome b c1 complex subunit 1, Uqcrh: Ubiquinone cytochrome b c1 complex subunit 6, Uqcrfs1: Ubiquinone -cytochrome C reductase iron-sulfur subunit Etfa: Electron transfer flavoprotein alpha subunit, Atp5e: ATP synthase subunit epsilon, Atp5a1: ATP synthase subunit ***α***, Acaa2: Acetyl-CoA acyltransferase 2, Acadl: Acyl-CoA dehydrogenase, Fabp3: fatty acid binding protein 3, Decr1: 2, 4 dienoyl CoA reductase, Hadh: Hydroxyacyl-CoA dehydrogenase, Ndufs4: NADH dehydrogenase [ubiquinone] iron-sulfur protein 4, Cox5a: Cytochrome C oxidase subunit 5A, Pdha/b: Pyruvate dehydrogenase A/B, Tnnt1: TroponinT, Myh10/11: Myosin heavy chain isoforms, Calm3: Calmodulin isofom 3, Gpx1: Glutathione peroxidase 1, Sod1/2: Superoxide dismutase type 1 or 2. Values represent the mean ± s.e.m. of data from 3 rats in each group. *P < 0.05 (AR versus GH, relative to control groups), these results were analyzed by 2-way ANOVA followed by multiple comparisons with Tukey’s test.

To understand molecular mechanisms for AR effect on cardiac function, we performed unbiased proteomic (LC – MS) analysis from heart tissue samples of control (untreated), AR and GH administered rats. Protein expression profile revealed that hearts of AR treated rats have increased expression of proteins regulating the tricarboxylic acid (TCA) cycle, oxidative phosphorylation (OXPHOS), fatty acid oxidation (FAO), cardiac muscle contraction, antioxidant defense and mitochondrial proteins (Fig. 3). LC-MS proteome profile of heart tissue of AR administered rats revealed significant (*P* < 0.05) increase in the expression of TCA driving enzymes (Sdha, Idh2, Cs); OXPHOS complex enzymes (Ndufs4, Etfa, COXIV, ATP5a1, Pdha), FAO (Fabp3, Hadh), muscle contraction (Tnnt1, Myh11, Calm3) and antioxidant defense (Gpx1, Sod1, Sod2) proteins (Fig. 3a and b).

**Figure 4:**
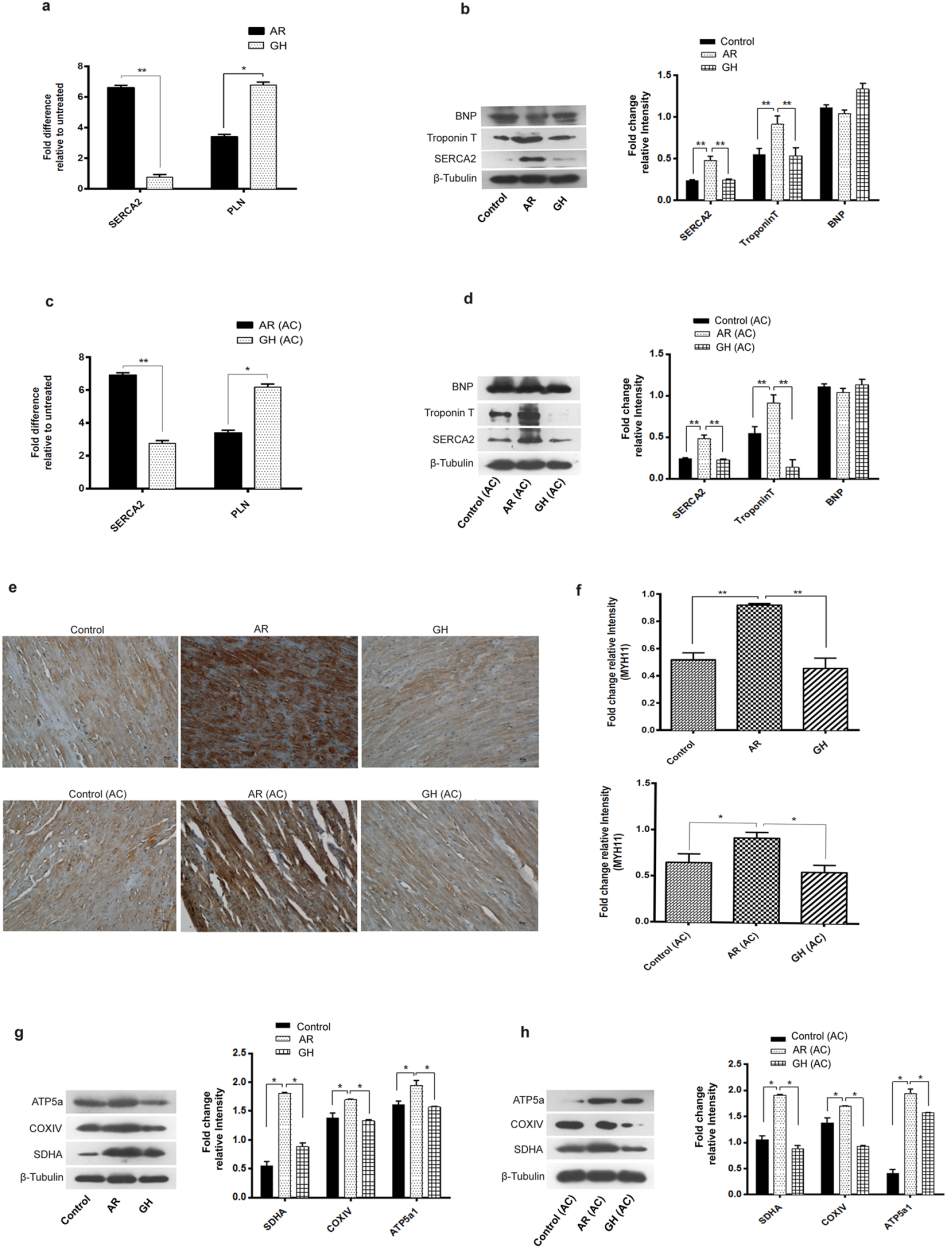
AR improved muscle contraction and mitochondrial bioenergetics function in aging and AC group of rats. (**a**,**c**) qRT-PCR analysis revealed an increase in mRNA expression of SERCA2 and decrease in PLN genes respectively in both aging and aorta constricted (AC) group of rats. (**b**,**d**) Representative immunoblots of BNP, troponinT, SERCA2a and β-tubulin from heart of rats and densitometry analysis revealed significant increase in expression of marker proteins which regulate the muscle contraction in both aging and aorta constricted (AC) group of rats. (**e**) Representative immunohistochemistry images of cross sections of hearts which were immunostained with Myh11 antibody (20X). (**f**) Quantification of immunostained myocardium revealed the increase in Myh11 expression in AR administered rats when compared with GH or control groups. (**g**,**h**) Representative immunoblots of ATP5a, COXIV, SDHA and β-tubulin from heart of rats and densitometry analysis revealed significant increase in expression of marker proteins which regulate the mitochondrial OXPHOS function, in both aging and aorta constricted (AC) group of rats. β-tubulin was used as loading control. Values represent the mean ± s.e.m. of data from 6 rats in each group. *P < 0.05, **P < 0.01 (AR versus GH or control; AR (AC) versus GH (AC) or control (AC) groups), results were analyzed by 2-way ANOVA followed by multiple comparisons with Tukey’s test.

**Figure 5:**
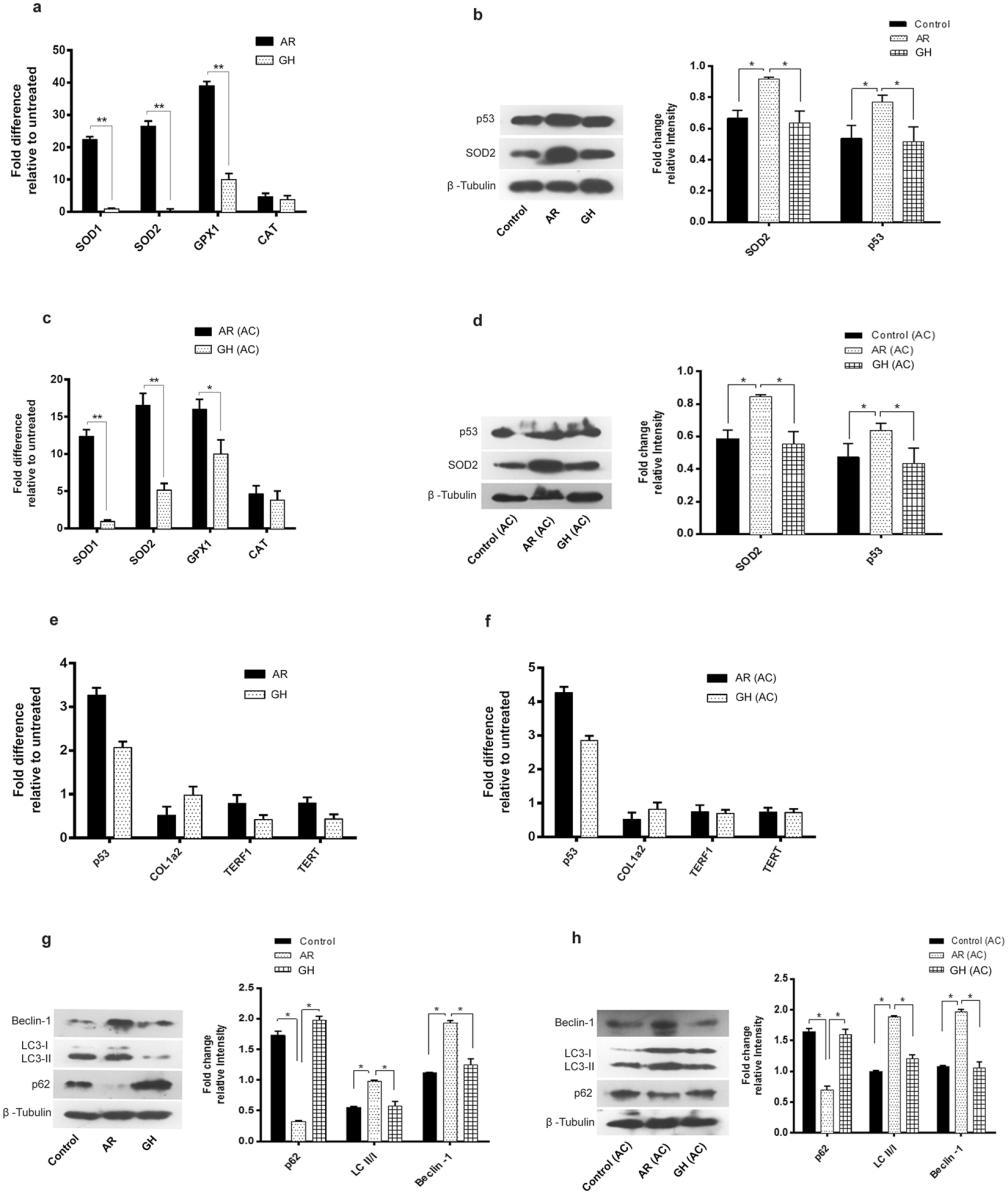
AR increased antioxidant defense and autophagy in both aging and AC group of rats. (**a**,**c**) SOD1 or 2, GPX1 and CAT mRNA expression were assessed by quantitative real-time reverse transcription-polymerase chain reaction and found a significant increase in the expression of these genes in AR administered rats. (**b**,**d**) Representative immunoblots of SOD2, p53 and β-tubulin from rat hearts and densitometry analysis revealed that SOD2 and p53 proteins were increased in AR administered rats. (**e**,**f**) p53, Col1a2, TERF1 and TERT mRNA expression were assessed by quantitative real-time reverse transcription-polymerase chain reaction. There was no significant difference in expression of these genes among the AR treated or untreated groups. (**g**,**h**) Representative immunoblots of Beclin-1, LC3-I/II, p62 and β-tubulin. β-tubulin was used as loading control. Values represent the mean ± s.e.m. of data from 4 rats in each group. *P < 0.05, **P < 0.01 (AR versus GH or control groups; AR (AC) versus GH (AC) or control (AC), results were analyzed by 2-way ANOVA followed by multiple comparisons with Tukey’s test.

These findings were further confirmed by qRT-PCR, Western blot and immunohistochemistry (IHC) analysis. Consistent with the proteomic results, SDHA, COXIV, ATP5a1, SERCA2, TroponinT, Myh11 (Fig. 4a–h) and SOD1, SOD2, GPX1 (Fig. 5a–d) were found to be significantly (*P* < 0.05) up regulated in AR administered rats while PLN and CAT1 were down regulated or unchanged respectively (Figs 4a,c and 5a,c). There were no significant differences among the hearts of different group of rats, in the expression of fibrosis related genes (p53, COL1a2, TERF1, TERT) which were analyzed by qRT-PCR (Fig. 5e,f).

**Figure 6:**
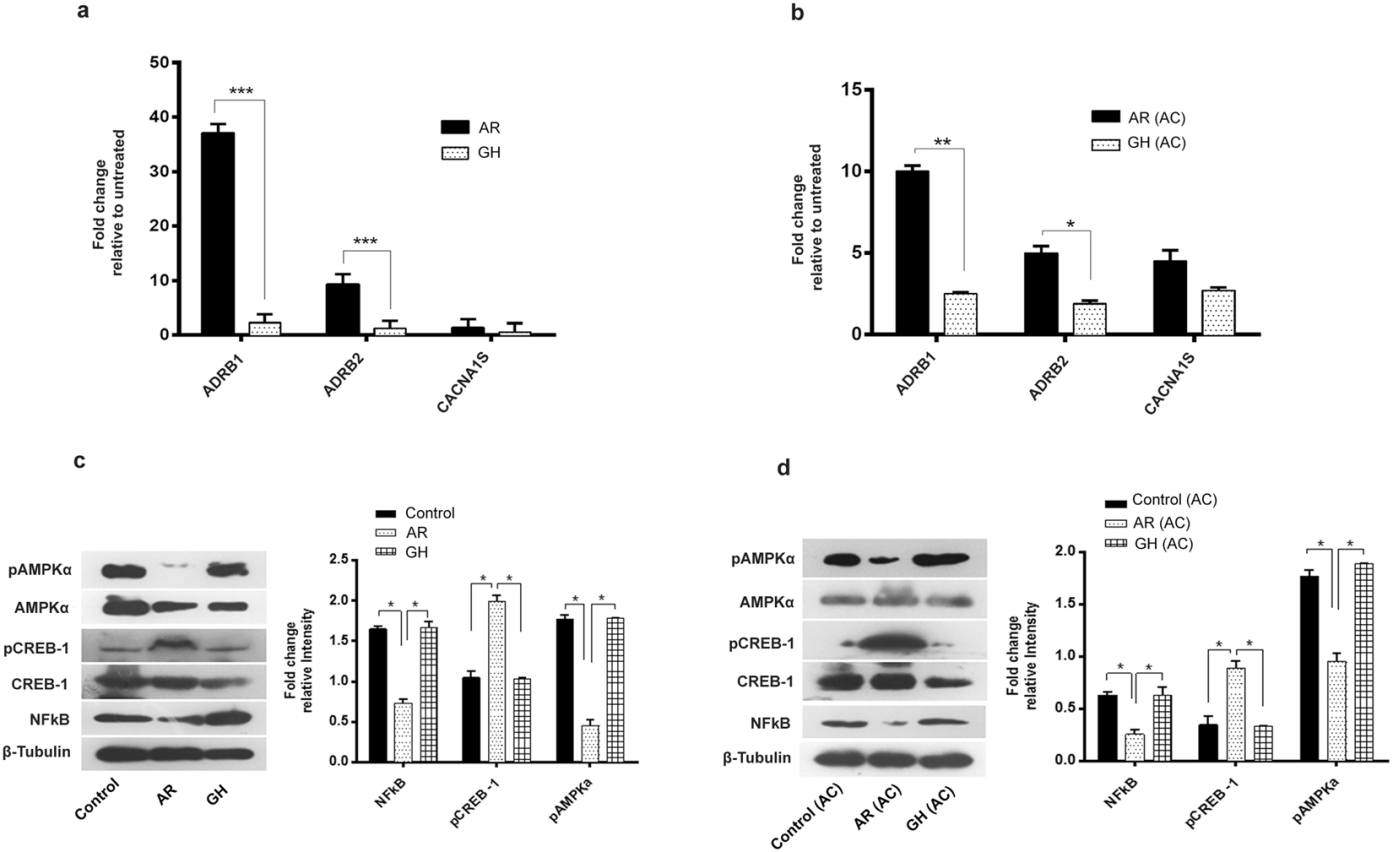
AR activates the ADRB1 or 2 (β-adrenergic receptors), pCREB and inhibit pAMPK in cardiac tissues of rats, both in aging and AC groups. (**a**,**b**) Significant increase in mRNA expression of ADRB1/2 genes while no change in voltage dependent Ca^2+^ channel (CACNA1S) in both aging and aorta constricted (AC) group of rats administered AR. (**c**,**d**) Representative immunoblots of pAMPK, AMPK, pCREB-1, CREB-1, NFkB and β-tubulin from rat hearts and densitometry analysis revealed increase in phosphorylation of CREB-1 and decreased phosphorylation of AMPK and down regulation in NFkB proteins were seen in AR administered rats of both aging and aorta constricted groups. β-tubulin was used as loading control. Values represent the mean ± s.e.m. of data from 4 rats in each group. *P < 0.05, **P < 0.01, ***P < 0.001 (AR versus GH or control groups; AR (AC) versus GH (AC) or control (AC), results were analyzed by 2-way ANOVA followed by multiple comparisons with Tukey’s test.

We assessed the conversion of LC3-I to LC3-II, a marker for autophagy induction, in heart tissues of AR administered rats and found that AR administration increased LC3-II accumulation (Fig. 5g,h). A significant (*P* < 0.05) increase in beclin-1 and decrease in p62 were also seen, suggesting autophagy induction in AR administered rats. We also observed a significant (*P* < 0.05) decrease in the levels of AMPKα phosphorylation in heart tissues of rats administered AR suggesting an energy efficient myocardium (Fig. 6c,d). A significant (*P* < 0.05) decrease in the expression of NFkB, a transcription factor which regulates stress response signaling in hearts of rats administered AR (Fig. 6c,d).

**Figure 7:**
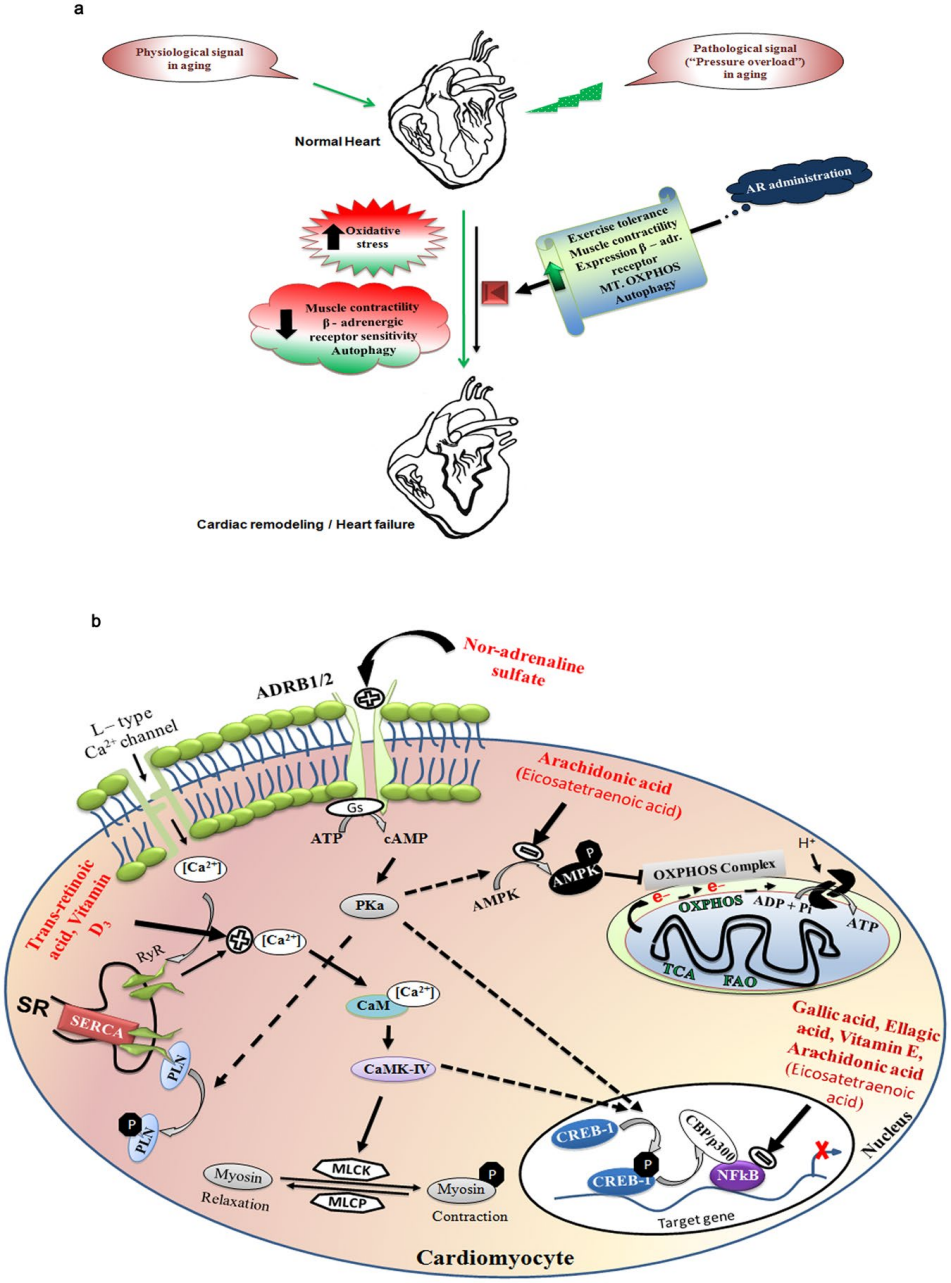
Possible molecular mechanisms of AR which improved the cardiac function in aged or aorta constricted group of rats. (**a**) AR administration attenuates the physiological and pathological signal (pressure overload hypertrophy) induced cardiac dysfunction in rats. (**b**) Nor-adrenaline sulfate activates the ADRB1/2 receptors which in turn activate CREB-1. Arachidonic acid, Gallic acid and Ellagic acid can inhibit pAMPK*α* or NFkB expression. Vitamin D_3_ and trans-retinoic acid regulate [Ca^2+^] in heart. pCREB-1, NFkB and pAMPK transcriptionally regulate muscle contraction, oxidative stress and mitochondrial bioenergetics function in heart respectively.

Since, AR administration improved ejection fraction, fractional shortening and exercise tolerance capacity in aging rats, we analyzed the expression of β - adrenergic receptor function and found increase in mRNA expression of ADRB1 and ADRB2 genes in cardiac tissues of AR administered rats (Fig. 6a,b). We checked the mRNA expression of CREB-1 (cAMP response element binding protein -1), a downstream signaling transcription factor activated on adrenergic stimulation and ADRB1/ADRB2 activation. Heart tissues of AR administered rats had significantly (*P* < 0.05) increased levels of CREB-1 (Ser133) phosphorylation (Fig. 6c,d). Increased pCREB-1 phosphorylation suggests that this factor may transcriptionally regulate the expression of observed changes in muscle contractile function and mitochondrial bioenergetics proteins. The possible mechanisms of AR and summary of its effects are suggested in Fig. 7.

A comparison of the effects of AR with some well known allopathic drugs, digitalis, nor-adrenaline, L-carnitine and Coenzyme Q are given in the Supplementary Table S7.

## Discussion

Current growing interest in the use of Ayurvedic medicine by specialists in modern medicine has spurred investigations on biologic effects of herbal drugs. The new science of Ayurvedic Biology focuses on creating evidence at organ, tissue, cellular and molecular levels for the beneficial effects observed at bodily levels in humans^16^. Our studies evaluated the effect of one of the important rasayana preparations in Ayurveda, known as Amalaki rasayana in ameliorating cardiac dysfunction associated with aging and pressure overload left ventricular hypertrophy.

Aging and pressure overload hypertrophy associated cardiac changes include impaired autonomic regulation, decreased cardiac contractility and reduced exercise tolerance^17, 18^. To reinstate the frail and compromised heart, new heart failure therapies have been explored to improve excitation-contraction and relaxation coupling, improving metabolism or activation of cell survival/autophagy pathways.

Our study in aging rats indicate that regular intake of AR improves left ventricular dimension, exercise tolerance capacity and left ventricular function. In rats with aortic constriction, AR administration improved left ventricular dimensions and exercise tolerance capacity. However, EF and FS were not improved in this group.

AR administration increases the expression of antioxidant defense and β_1/2_ – adrenergic receptor genes in the rat heart. Our results indicate that increased mitochondrial OXPHOS, FAO and autophagy contribute to improvement in cardiac function in AR administered rats. AR is not toxic to myocardial cells as observed in our cytotoxicity assay. We have identified metabolites such as gallic acid, ellagic acid, vitamin A, 1alpha 24R,25-trihydroxyvitamin D3, 13’-carboxy-alpha-tocotrienol (Vitamin E), sulfate derivative of norepinephrine and putative arachidonic acid derived anti-inflammatory metabolites, in AR. Previous studies have reported that these metabolites play central roles in the regulation of myocardial bioenergetics, contractile, myocardial inefficiency and dysfunctional excitation-contraction coupling and hemodynamic function^19–23^. Presence of these components in AR could possibly contribute to improvement in cardiac function in rats administered with AR. We have observed gallic acid and ellagic acid as the major components of AR; vitamin C was present in only relatively small amounts in AR. In a recent study, Vishwanatha *et al*., also reported the presence of gallic acid as the major component (>42% abundance) of Amalaki rasayana. Vitamin C was only 1.8% in AR. They found that AR administration stably maintained the double strand break repair (DSBR) without altering the nucleotide excision repair or base excision repair in aged human individuals^3^. Gallic acid, ellagic acid and vitamin E are known to increase the antioxidants status in the body^24, 25^. Abundance of these components in AR could lead to increase in the antioxidant enzymes SOD1 or 2 as well as Gpx and decrease in NF-kB expression. Both Vitamin A and D_3_ (especially in combination) have been shown to attenuate cardiac remodeling associated changes with hypertrophy^26^. Presence of these components in AR could have contributed to decrease in degrees of hypertrophy and fibrosis or increase in anti-hypertrophy markers (SERCA2, TroponinT, Myh11) in AR administered animals. Nor-adrenaline sulfate present in AR may contribute to increase in ADRD1/2, pCREB-1 or exercise tolerance as reported previously^27, 28^. Arachidonic acid metabolite (5(S), 6(S)-epoxy-15(S)-hydroxy-7E, 9E, 11Z, 13E-eicosatetraenoic acid), play important roles in optimal metabolism and in reducing heart disease risk^29, 30^. This component in AR possibly aids in maintaining regulation of OXPHOS pathway, pAMPK, antioxidant enzymes status and decrease in NF-kB expression. The effects observed after AR treatment may be due to synergistic effects of the components. The doses of individual components in AR are small and we have not explored whether these small doses of each component separately would produce the beneficial effects.

Previous studies have shown cardiac structural changes associated with aging. These changes consist of increase in cardiomyocyte size, increased septal thickness and increased internal dimension of the left ventricle. They contribute to contractile dysfunction in the aging heart^17, 31, 32^. We found significant improvement in LVEF and LVFS in the aging animals and decrease in IVSd and LVPWd in animals with left ventricular hypertrophy secondary to aortic constriction which were administered AR. Age related decline in exercise tolerance and physical deconditioning are well established^33, 34^. AR significantly improved the fatigue time in treadmill exercise in aging rats and rats with severe left ventricular hypertrophy.

We have explored the molecular mechanisms of how AR possibly contributes to improvement in cardiac function. Previous studies suggest that altered mitochondrial dynamics or reorganization of respiratory chain complexes, oxidation of mitochondrial proteins and reduced biogenesis contribute to decline in efficiency of mitochondrial bioenergetics associated with aging^35–37^. AR is found to enrich OXPHOS, FAO, mitochondrion regulatory proteins and up regulate expression of OXPHOS complexes such as SDHA, COXIV, and ATP5a1 in cardiac tissues.

AMPK is a nutrient sensor transcription factor which regulates cellular energy metabolism and aging through multiple mechanisms. AMPK activation shuts off mTORC, indicates a high -AMP/ATP-low stage in aged myocardium and contributes to energy inefficiency^38, 39^. In cardiac tissues of AR administered rats, phosphorylation levels of AMPKα, an indicator of sufficient OXPHOS function was decreased, which suggests that these rats have energy efficient hearts.

The free radical theory of aging proposed that gradual decline in mitochondrial function with aging contributes to impaired electron transport chain (ETC) function and reduced ATP generation. Deficient ETC results in increased production of ROS, which in turn intensifies the mitochondrial dysfunction and overall cell damage^37, 40^. In our study, p53, SOD1, SOD2, GPX1 levels were significantly increased in the hearts of AR administered rats. These changes indicate improved antioxidant defense upon AR administration. Nuclear factor –kappaB (NFkB), a redox sensitive transcription factor is known to be up regulated during stress response signaling and in turn reduces the expression of SOD1 and SOD2 genes in the mitochondria^41, 42^. Expression of NFkB was decreased in the hearts of AR administered rats. This observation further strengthens the possibility that AR improves the antioxidant defense in aged heart.

Several studies have shown that either accumulation of fragmented mitochondria or decline in clearance of damaged organelle (autophagy) or both, contribute to increased mitochondrial degeneration which drives the pathological changes during aging^37, 43, 44^. It is widely known that beclin-1 is required for initiation of the autophagosome formation. LC3II accumulation indicates autophagy induction while p62 is degraded by autophagy process^45^. There was increased expression of autophagy markers LC3 and beclin-1 as well as decrease in the expression of p62 (marker for autophagy induction) in the hearts of AR administered rats, which suggests the effect of AR on a healthy cellular or tissue aging process.

AR also seems to improve the muscle contractility in the aged heart. Impaired cardiac muscle excitation – contraction coupling, decreased ATP dependent sarcoplasmic reticulum Ca^2+^-transport or SERCA activity, altered expression of calmodulin (Ca^2+^ binding protein), and sarcopenia are the reported critical changes during cardiac remodeling during aging^46, 47^. Increased expression of SERCA2, Calm3, TroponinT, Myh11, and decreased expression of PLN genes were seen in the hearts of AR administered rats. The increased muscle contractile performance could be the contributory factor in increased fatigue time in the AR administered rats.

Human and animal studies have revealed a decline in beta –adrenergic receptor sensitivity, or receptor density which contribute to decrease in cardiac contractility, SR dependent Ca2+ signaling and ejection fraction in the aged heart^17, 18, 48^.We analyzed the expression of selected membrane receptors (β_1/2_ adrenergic receptor (ADRB1/2), voltage dependent Ca^2+^ channel (CACNA1S), Na^+^-Ca^2+^ exchanger/NCX) whose activation can regulate Ca^2+^ regulation, muscle contraction or metabolic activity in the hearts of rats in our experiments. Increased expression of β_1/2_-adrenergic receptor especially ADRB1 gene, was seen in the hearts of AR administered rats which indicates activation of these receptors. An increased phosphorylation of CREB-1, a downstream target of ADRB activation was also seen.

Our study thus throws light on the possible mechanisms by which AR intake results in improved exercise tolerance. These mechanisms include improved mitochondrial bioenergetics through transcriptional regulation of mitochondrial OXPHOS and enhanced muscle contractility through activation of endogenous β-adrenergic receptor 1 and 2 (β-AR). The effects of AR seem to have similarities to those of standard Western drugs such as digitalis and nor-adrenaline and adjunctive agents such as L-carnitine and Coenzyme Q^49–51^.

Our study has some limitations. AR was administered to animals for only 5 days a week. A continuous administration for 7 days could produce a different response. For preparation of AR, we have followed the procedure specified in the Ayurvedic Text Charaka Samhita. The rationale for repetition of some of the steps in the procedure such as trituration, are unknown and needs further validation. Also, we did not perform pharmacokinetic analysis for AR. Previous pharmacokinetic analysis of gallic acid, the major component of AR has revealed the 4-O-methylgallic acid (4OMGA) as the major metabolite in human plasma^52^. Mertens-Talcott *et al*., (2006) reported urolithin A, hydroxyurolithin A, urolithin A-glucuronide, urolithin A and dimethyl ellagic acid-glucuronide as metabolites of EA in human urine samples^53^. Cardiac effects of these metabolites are unknown.

In summary, our data suggest that AR could be an important adjuvant for improvement of cardiac muscle contractility and mitochondrial energy efficiency in aging and hypertrophied heart.

## Materials and Methods

All animal experiments were carried out with the approval of the Institutional animal ethics committee (IAEC) in Rajiv Gandhi Center for Biotechnology (RGCB) under the protocol no. IAEC/150/CCK/2012. Animal experiments were conducted by strictly following the rules and regulations of the Committee for the Purpose of Control and Supervision of Experiments on Animals (CPCSEA), Government of India.

### AR (Amalaki rasayana) preparation protocol

*Amalaki rasayana* and GH were prepared and supplied by Arya Vaidya Sala, Kottakkal, Kerala, India. The fresh green Amalaki was procured from traders who source it from the Sathyamangalam area of Tamil Nadu (11.5048° N, 77.2384° E). The dried variety was procured from traders in Madhya Pradesh who, in turn, source it from Chattisgarh (21.2787° N, 81.8661° E) and from Madhya Pradesh (22.9734° N, 78.6569° E). Fresh gooseberries used for preparation of juice were harvested in November, 2012 and March, 2013. Gooseberries used to prepare the powder were harvested in November, 2011. The procedure used for preparation is given below and has been described earlier^9^.

**Step 1**. Dried gooseberry fruits (*Phyllanthus* *emblica*) were pulverized by a Tyco pulverizer to obtain powder (80 mesh).

**Step 2**. Fresh gooseberries were crushed by using a juice extractor to obtain the juice.

**Step 3**. The powder (product of step1) was blended in the gooseberry juice (product of step 2) in 1:1 ratio and dried for 24 hours at 55 °C under 700 mmHg by using a vacuum tray drier. The dry mass was then pulverized.

Steps 2 and 3 were repeated 20 times. The 21 times repeated step is called in Ayurvedic texts as *bhavana* (trituration). This is a procedure prescribed for the purpose of potentiating the basic activity of the ingredient herb and it involves mixing the powder with a juice medium, then drying it and again going through the cycle repeatedly. This is the standard procedure recommended in the preparation of *Amalaki rasayana* ^54^.

**Step 4**. The powdered mass of amalaki (obtained after completion of 21 trituration cycles) was blended with honey (M/s. Galaxy Honey, Kottakkal, Kerala) and Ghee (“Milma” from Malabar Milk Marketing Federation, Govt. of Kerala, Kozhikode, Kerala) in a 1: 2:0.5 ratio. Finally, a thick pasty mass of Amalaki rasayana was obtained as described previously by Dwivedi *et al*., 2012^8^. Composition of ghee used is provided in the Supplementary Table S8.

The ancient Ayurvedic text ‘Charaka Samhita’ mentions method of preparation of AR and we have followed the method prescribed in this text. Honey and ghee are added to the triturated Amalaki powder as ‘anupana’ (carrier or adjuvant), which is considered as a medium to improve acceptability and palatability and also to help absorption of the main ingredient^54, 55^.

GH was constituted with a mixture of ghee and honey as (mixed in a fixed proportion (2: 0.5)) used in the preparation of Amalaki rasayana.

Quality assurance and control assays were performed by standard protocols as described by Dwivedi *et al*., 2012. Briefly, Centre for Medicinal Plants Research, Kottakkal and the Quality Assurance (QA) department of Arya Vaidya Sala, (approved for testing and issuing quality control certificates for Ayurvedic raw materials and finished products by Govt. of India) authenticated the Amala fruit and powder.

Ayurvedic Pharmacopoeia of India (API) certified that macroscopic (shape and taste of fruits) and microscopic (nature of pericarp, mesocarp and vascular bundles of fruits) characteristics of Amala were equivalent with the specified standards (http://www.ayurveda.hu/api/API-Vol-1.pdf)^56^. Ayurvedic Pharmacopoeia of India contains the Official Standards to be adapted and followed for Ayurvedic preparations. HPTLC profiles of raw Amalaki fruits, Amalaki powder, samples at the conclusion of 1st, 10th, 15th and 21st steps of trituration and the finished formulation revealed the presence of gallic acid and ellagic acid compared with the respective standards.

### Characterization of components of Amalaki rasayana (AR)

#### Solubility analysis

To determine the solubility of AR and GH, the following solvents were used, (i) acetonitrile: water (20:80 v/v) and (70:30 v/v), (ii) methanol: water (20:80 v/v) and (70:30 v/v), (iii) ethanol: water (20:80 v/v) and (70:30 v/v) and diethyl ether:water (20:80 v/v) and (70:30 v/v). The analysis was performed by adding 10 mg of each preparation in different solvents to observe the maximum solubility. 20% ethanol in water (20:80 v/v), was observed as a good solvent for both AR and GH. Further, after assessment of solubility, RP-HPLC profile was analyzed for both AR and GH.

#### RP-HPLC profile of AR and GH

AR and GH were analyzed using Waters HPLC equipped with symmetry C18 column (150 mm × 3.9 mm i.d., 5 μm), 2707 auto sampler and 2489 UV/Visible detector. Breeze software (Waters, USA) was used for monitoring and processing output signal. Elution was done at a flow rate of 1 ml/min using mobile phase comprising of Solvent A [Water: acetic acid (ratio of 99.9:0.1 v/v)] and solvent B as acetonitrile. The samples were eluted by gradient mentioned in Table 1. The column temperature was maintained at 25 °C, detection wavelength was set at 265 nm and injection volume of 25 μL was used.

The relative hydrophobicity of elution system shifts from more hydrophobic compounds to less hydrophobic compounds. Thus, for further identification of components of AR, LC-MS analysis was done.

#### LC-MS analysis of AR

Step 1: One gram of AR was dissolved in 20 ml (20% ethanol in water) in a conical flask at 4 °C under overnight stirring. Step 2: From the solution, 10 μl volume was loaded on TLC (Thin Layer Chromatography, TLC Silica gel 60 F_254_) plate and separated using the mobile phase Toluene: ethyl acetate: formic acid: Methanol (6: 6: 1.6: 0.4) ratio. Step 3: after TLC fractionation of AR, three different components (depends on the polarity of components named as C1, C2 and C3) were separated on silica plate. Step 4: separated components were scraped from plate under UV light, and all the three components were dissolved in methanol (100%) and were lyophilized. Step 5: the lyophilized fractions (dissolved in acetonitrile) were given for LC-MS analysis using the instrument (Synapt-G2, Q-TOF LC-MS/MS from waters). MassLynx and PLGS v2.5.3 were used as software for data acquisition and for raw data processing respectively. Step 6: after getting the data from the LC-MS, we uploaded the data in XCMS online software for metabolite identification.

### Assessment of cytotoxicity of AR

#### Cell culture

Rat embryo’s heart ventricular cells (H9c2 cardiomyoblast) were obtained from American Type Culture Collection. The cells were maintained in DMEM (95%), Fetal bovine serum (5%), Penicillin/Streptomycin (U/ml) at 37 °C in 5% CO_2_. Fetal bovine serum concentration was reduced to 1% twelve hours before treatment with AR.

#### Cytotoxicity assay

(MTT/3-(4,5 - dimethylthiazol-2-yl)-2,5-diphenyltetrazolium bromide) assay. To assess the safety/toxicity profile of AR, MTT assay was performed in H9c2, the rat cardiomyoblast cells. Cells were sub cultured in 96-well microtiter plates at a density of 5 × 10^3^ cells per well at 37 °C in CO_2_ incubator. After overnight incubation, cells were incubated with or without AR in different doses (10 μg, 100 μg, 1 mg, 10 mg, and 100 mg/ml dissolved in phosphate buffered saline) for 24 h, 48 h, or 72 h. After completion of incubation time, the medium was removed and 20 μL of MTT (5 mg/mL in PBS) was added with fresh medium. To dissolve the formazan crystal formed, 100 μL of isopropanol was added with mild agitation at room temperature for 1–2 minutes. Spectrophotometric absorbance was measured at 570 nm in an ELISA microplate reader (BioRad). The percentage of cell viability was calculated by the following formula: Viable cells % = (*OD* of AR-treated cells/*OD* of untreated (MTT + Isopropanol only) cells) × 100.

### Animal experiments

Three-month- old male Wistar rats, (180–200 g) were used. The animals were housed in 12-hours day/light cycle with temperature and humidity controlled room. Rats were fed with rodent synthetic chow diet and had *ad libitum* access to water.

#### Rat model of cardiac hypertrophy

Rats were anesthetized with 3% isoflurane with 100% O_2._ To induce ‘pressure overload’ cardiac left ventricular hypertrophy, the ascending aorta was constricted (AC) using a titanium clip (constricted approximately 60% of original diameter)^51^. Pressure gradient was recorded using trans- thoracic two dimensional color Doppler analysis to ensure physiologic constriction of the aorta. Sham operated rats underwent similar surgical procedure as in rats with constriction of aorta except for constriction of aorta. Left ventricular hypertrophy was consistently observed during echo studies in rats after 8 weeks of aortic constriction.

#### Experimental design

Two sets of experiments (Experimental design is given in supplementary Fig. S2A & B) were conducted: (i) in aging group of rats and (ii) in rats with left ventricular hypertrophy (LVH), induced by constricting the ascending aorta with titanium clips.

In experiment I (aging groups), 56 male rats (3 months of age) were divided into seven groups; three groups (each group having 8 rats each) were given AR (either 750 mg/kg or 500 mg/kg or 250 mg/kg body weight, orally 5 days a week). Another 3 groups (each group having 8 rats each) were given GH (750 mg/kg or 500 mg/kg or 250 mg/kg, orally 5 days a week) while the seventh group was left untreated as controls (n = 8). All these rats in the 7 groups were allowed to grow up to 21 months of age. Since effects of AR on cardiac function has not been investigated previously, we tried three different doses (250, 500 and 750 mg/kg body weight). We found 500 mg/kg body weight dosage optimally effective.

This dose was considered for experiment 2. 500 mg/kg body weight dosage was selected as an optimum dose also because of the traditional use in humans in Ayurveda practice and also based on previous animal studies^13^. In experiment II (ascending aorta constricted–AC groups), ascending aorta was constricted using titanium clips to induce left ventricular hypertrophy in 24 rats (3 months old). After the rats developed left ventricular hypertrophy, AR (500 mg/kg, orally 5 days a week, N = 8) or GH (500 mg/kg body weight, orally, N = 8) was given to 2 groups of rats. Another 8 rats (AC) did not receive either AR or GH.

Our analysis of AR revealed that a 500 mg/kg body weight dosage of AR would have 7.5 mg gallic acid (GA) and 2 mg ellagic acid (EA) as daily dose. All the animals were observed daily for food and water intake as well as for any untoward symptoms. Body weights were regularly recorded.

#### Drug administration

AR or GH was administered orally to the aging group of rats each day morning for 5 days a week until 21 months of age. The rats in AC group were administered AR or GH orally each day for 5 days a week, for 12 months after the rats developed left ventricular hypertrophy (9 months of age) as assessed by echocardiography. The experiment was continued until the AC group rats attained 21 months’ age.

#### Echocardiography

To investigate the effect of AR on age associated changes in left ventricular dimensions and ejection fraction associated with aging^17, 31, 32^ we performed echocardiography studies. Echocardiography was performed in all the rats at 3 months of age before starting the experiments (prior to administration of AR), 12 months of age (9 months after start of AR administration) and 21 months of age (18 months after start of AR administration) in the aging group. In AC group, echocardiography was performed in all the rats before the start of the experiment (3 months of age) and after constriction of aorta at intervals of 3, 6 (6 and 9 months of age respectively) and 21 months (12 months after initiation of administration of AR).

#### Exercise tolerance

One month prior to sacrifice of animals, all the rats were trained on a motorized treadmill (Columbus) with individual Plexiglas lanes for 30 min, 5 days/week. During the first week of training, animals were acclimatized to the treadmill by gradually increasing the running speed (5, 10, 15 m/min), without inclination while frequency and number of shocks were fixed each time at gradually increasing speeds. At the end of two weeks of training, exercise tolerance was assessed by following the standardized protocol (with motorized belt speed of 5, 10 and 15 m/minute). Fatigue time versus distance travelled was measured in triplicate for all rats (N = 8, each group) in both the aging and AC groups.

*Assessment of heart failure* was carried out at the end of the experiment, by estimation of serum levels of brain natriuretic peptide (BNP). BNP assay was done following the manufacturer’s protocol with BNP -32 rat ELISA kit (Abcam).

#### Collection of tissue

Animals were sacrificed by euthanasia and the hearts were collected.10% neutral buffered formalin was used for fixing the 5 mm thick cross sections above the apex and processed for paraffin embedding following standard protocol. Tissues were also collected from the heart for proteomic profiling by Liquid chromatography and mass spectrophotometry, qRT-PCR and Western blot. These tissues were collected in RNAlater, kept at room temperature for 12 hours and then stored in −80 °C until analysis.

#### Histology

From the paraffin block of heart tissues, 5 μm thick sections were cut on a microtome (Leitz). Hematoxylin and Eosin and Masson trichrome (HT10516-500 ML, SIGMA ALDRICH, USA), stained sections were examined under a Nikon Eclipse 55i microscope for assessment of myocyte and vascular changes and fibrosis respectively. To quantify the cardiomyocyte diameter and fibrosis, we have used software NIS element (Nikon). Hypertrophy was measured by calculating the HW/BW (mg/g) ratios and cardiomyocyte dimensions. Minor cell diameter (D_min_, μm) and major cell diameter (D_maj_, μm), comparable to the cardiomyocyte length, were measured as parameters to quantify the cardiomyocyte size from trichrome stained sections. Longitudinally cut cardiomyocytes were selected for D_maj_ and multiple measurements were made from each of the anterior, septal, lateral, and inferior wall in LV sections. Cross sections of cardiomyocytes were taken to measure D_min_. Well defined cell membrane and visible nuclei were the criteria for selection of myocytes (Fig. 2a).

Fibrosis score (semi-quantitative scale, 0–4) and fibrosis (area %) were calculated based on the analysis of perivascular and interstitial fibrosis in 15 microscopic fields from each section of left ventricle (n = 6) by using software NIS element from Nikon 55i microscope. Semi-quantitative scores of 0, 1, 2 and 3/4 were assigned for normal, mild, moderate and severe fibrosis. Percentage fibrosis was calculated as percent relative blue staining to total myocardial area from the quantified RGB images.

#### Immunohistochemistry

Sections from paraffin embedded tissue blocks were deparaffinized and rehydrated. After retrieval of antigenicity in tissue sections, Super Sensitive Polymer-HRP IHC Detection System/DAB (QD400-60 K, BioGenex Life Sciences Private Limited, India) kit was used for further steps. Mouse monoclonal antibody to smooth muscle myosin heavy chain 11 (ab683) was used in 1:100 dilutions with 3% BSA in PBS. After washing with PBS, sections were incubated for 1 hour with secondary antibody conjugated to horse radish peroxidase (HRP). Then the sections were stained with hematoxylin (Sigma-Aldrich) for nuclei and mounted in DPX (Di-N-Butyl Pthalate in xylene, Merck). Intensities of immunostained images were quantified by using Nikon NIS elements software (Japan). The relative intensity of staining in the myocardium was corrected for background, normalized with GAPDH and expressed as fold change relative to untreated samples (N = 6).

### Proteomic profiling

#### Protein sample preparation

Heart tissues were ground, homogenized with a pestle and mortar in liquid nitrogen and the lysate was prepared in 0.3% RapiGest^TM^ SF surfactant in 50 mM NH_4_HCO_3_ buffer (Waters, USA). Total protein content was estimated by Bradford assay. Peptide was generated for each sample (100 µg of protein) using in-solution trypsin digestion. Solutions of digested peptide were centrifuged at 14000 rpm for 12 minutes and supernatant was stored at −20 °C until LC/MS/MS analysis.

#### Liquid Chromatography and Mass Spectrometry

Peptide samples were analyzed by using a nano ACQUITY UPLC® System (Waters, UK) coupled to a Quadrupole-Time of Flight (Q/TOF) mass spectrometer (SYNAPT-G2, Waters, UK) controlled by MassLynx4.1 SCN781 software (Waters, UK). Peptides eluted from the nano LC were monitored by SYNAPT® G2 High Definition MS™ System (HDMSE System (Waters, UK). Three technical replicate runs were performed for each sample.

#### Data analysis and bioinformatics

LC/MSE data were analyzed by using ProteinLynx Global SERVER™ v2.5.3 (PLGS, Waters, UK) which employs protein identification as well as relative quantification. Database search was performed using *Rattus novergicus* database from NCBI. Protein identification was performed by setting the parameters for each peptide such that at least one fragment ion match and a protein was required to have at least three fragment ion matches, or at least two peptide matches for identification. Precursor and fragment ions were defined by setting the Mass tolerance at 10 and 20 ppm respectively. Oxidation of methionine and carbamido-methylation of cysteine were chosen as variable modification and fixed modification respectively. Auto-normalization of PLGS was done to normalize the dataset and label-free quantitative analyses were carried out and compared with normalized peak area/intensity of identified peptides between the samples. Number of peptides, score, and sequence coverage parameters were identified for each protein. The reference sequence identifications (RefSeq.) obtained after the PLGS analysis was converted into gene symbols by Biological Database Network (BioDBnet). Gene symbols were categorized into different biological functions by using a bioinformatics tool, **D**atabase for **A**nnotation, **V**isualization and **I**ntegrated **D**iscovery (DAVID). Statistical analysis and graphical representations were performed through MS-Excel 2010 and GraphPad Prism (6.0).

**qRT-PCR** (Quantitative reverse transcription polymerase chain reaction): Heart tissues were homogenized in Trizol (Life technologies 15596-018) with a pestle and mortar. After confirmation of the quality, RNA was reverse transcribed into complementary DNA followed by gene amplification through qRT-PCR using the software SDS in Bio-Rad RT-PCR machine. After confirmation of primer efficiency in all the experiments, primer specificity (single product amplification) was analyzed by melting-curve analysis. Target mRNA expression was quantified, normalized to the GAPDH and fold change calculated by 2^−ΔΔCt^ method. mRNA expression results were represented as fold difference in target gene amplification in tissues of AR/GH administered rats, relative to amplification in tissues of untreated rats (N = 6). Details of primers used are provided in Supplementary Table S9.

**Immunoblot analysis:** Heart tissues excised from the rats during sacrifice were snap - frozen in liquid N_2_ and stored at −80 °C. Heart tissues were ground homogenized with a mortar and pestle in liquid nitrogen and then lysed with Radioimmunoprecipitation assay buffer (RIPA buffer) using a dounce homogenizer. 40 μg of total protein was loaded for Immunoblot analysis from each tissue lysate. The prepared tissue lysates were processed for Immunoblot analysis. The relative intensity of band in blot was corrected for background, normalized with β-tubulin and expressed as fold difference (N = 6, *P* < 0.05) in AR, GH administered or control samples relative to the housekeeping protein (β-tubulin). Details of antibodies used are provided in Supplementary Table S10.

### Statistical analysis

Data were analyzed by software GraphPad Prism (6.0). Results are presented as mean ± SEM unless otherwise indicated. Multi-comparison of data was performed through either a repeated measure one-way ANOVA, one-way ANOVA followed by multiple comparisons with Tukey’s test, repeated measure two-way ANOVA, two-way ANOVA followed by multiple comparisons with Tukey’s test, as indicated in legends for respective figures or tables. We have considered *p* < 0.05 as statistically significant.

## Electronic supplementary material

Supplementary Information
