# Supplementary material for: Amalaki rasayana, a traditional Indian drug enhances cardiac mitochondrial and contractile functions and improves cardiac function in rats with hypertrophy
Source: Sci Rep. 2017 Aug 17;7:8588. doi: 10.1038/s41598-017-09225-x (PMC5561106; doi:10.1038/s41598-017-09225-x)
Supplement: Supplementary file 1 — Supplementary Information [file 41598_2017_9225_MOESM1_ESM.pdf]

***Amalaki rasayana,* a traditional Indian drug enhances cardiac mitochondrial and contractile functions and improves cardiac function in rats with hypertrophy**

Vikas kumar[1], Aneeshkumar A[1], Kshemada K[1], AjithKumar GS[1], Binil Raj SS, Neha Deora[3], Sanjay G[2], Jaleel A[1], MuraleedharanT.S[4], Anandan E.M[4], Mony R.S[4], Valiathan MS[5], Santhosh Kumar TR[1], Kartha C.C[1*].

[1]Cardiovascular Diseases and Diabetes Biology, Rajiv Gandhi Centre for Biotechnology, Trivandrum, India

[2]Department of Cardiology, Sree Chitra Tirunal Institute for Medical Sciences & Technology, Trivandrum, India

[3]Centre for Bio-Separation Technology, Vellore Institute of Technology, Vellore, India

[4]Kottakal Arya Vaidyasala, Kottakkal, Kerala, India

[5]Manipal University, Manipal, Karnataka, India

Contact information. Dr. CC. Kartha, MD, Distinguished Professor, Cardiovascular and Diabetes Biology Group, Rajiv Gandhi Centre for Biotechnology, Thycaud Post, Poojappura, Trivandrum – 695014, INDIA

E. mail: cckartha@rgcb.res.in

Phone: +91-471-2529484

Supplementary figure legends.

Supplementary Fig. S1. Characterization of AR preparation revealed the abundance of components which have biological importance in cardiovascular system. (a) HPTLC profile of finished AR preparation**. (b)** RP-HPLC profile AR in ethanol. **(c)** RP-HPLC profile of AR in acetonitrile. (**d)** RP-HPLC profile of GH in ethanol. **(e)** RP-HPLC profile of GH in acetonitrile. **(f)** Pie diagram of whole components from AR (LC-MS followed by XCMS software analysis). **(g)** Comparison of viability of H9C2 cells treated with AR (dissolved in phosphate buffered saline)**.** Percentage cell viability was calculated as Viable cells % = (*OD* of AR-treated cells/*OD*

of untreated (MTT + Isopropanol only) cells) × 100. Values represent the mean ± s.e.m. of data from three experiments.

Supplementary Fig. S2. Summary of experimental design. (**a)** After 4 weeks on synthetic chow diet for acclimatization, 3 months old rats had baseline echocardiography and were administered with AR (250, 500, 750mg/kg, orally/day) or GH (250, 500, 750mg/kg, orally/day) and control (untreated). After recording the 2nd and 3rd echo, animals were sacrificed. (**b)** After 4 weeks on synthetic chow diet for acclimatization, 3 months old rats had baseline echocardiography and ascending aorta was constricted for inducing left ventricular hypertrophy. After assessment of hypertrophy, rats were administered with AR (500 mg/kg, orally/day) or GH (500mg/kg, orally/day) and control. After 12 months of treatment, tissues were harvested.

Supplementary figures are given below,

Supplementary Fig. S1

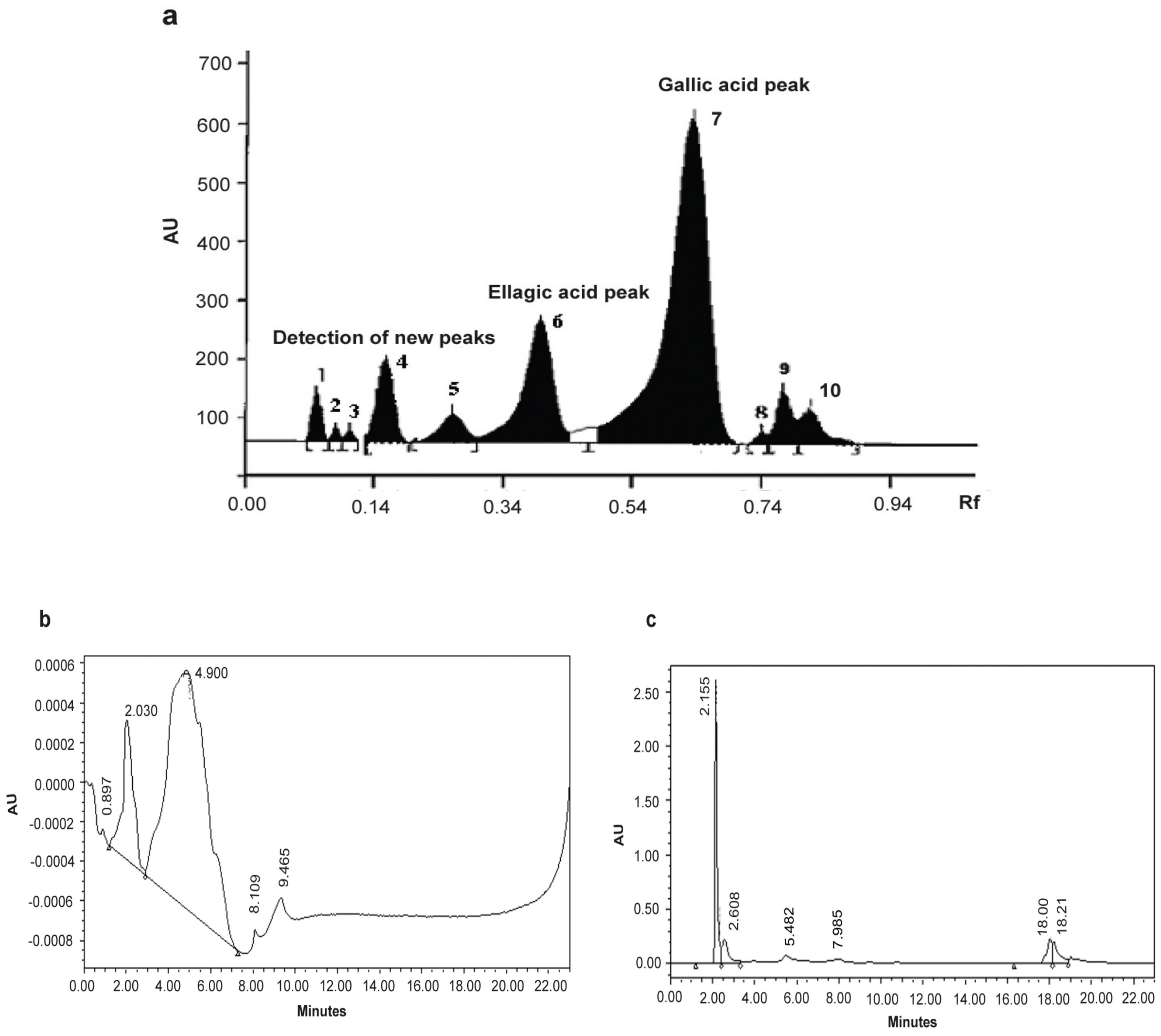
a
Gallic acid peak
Ellagic acid peak
Detection of new peaks
AU
Rf
b
AU
Minutes
0.897
2.030
4.900
8.109
9.465
c
AU
Minutes
2.155
2.608
5.482
7.985
18.00
18.21

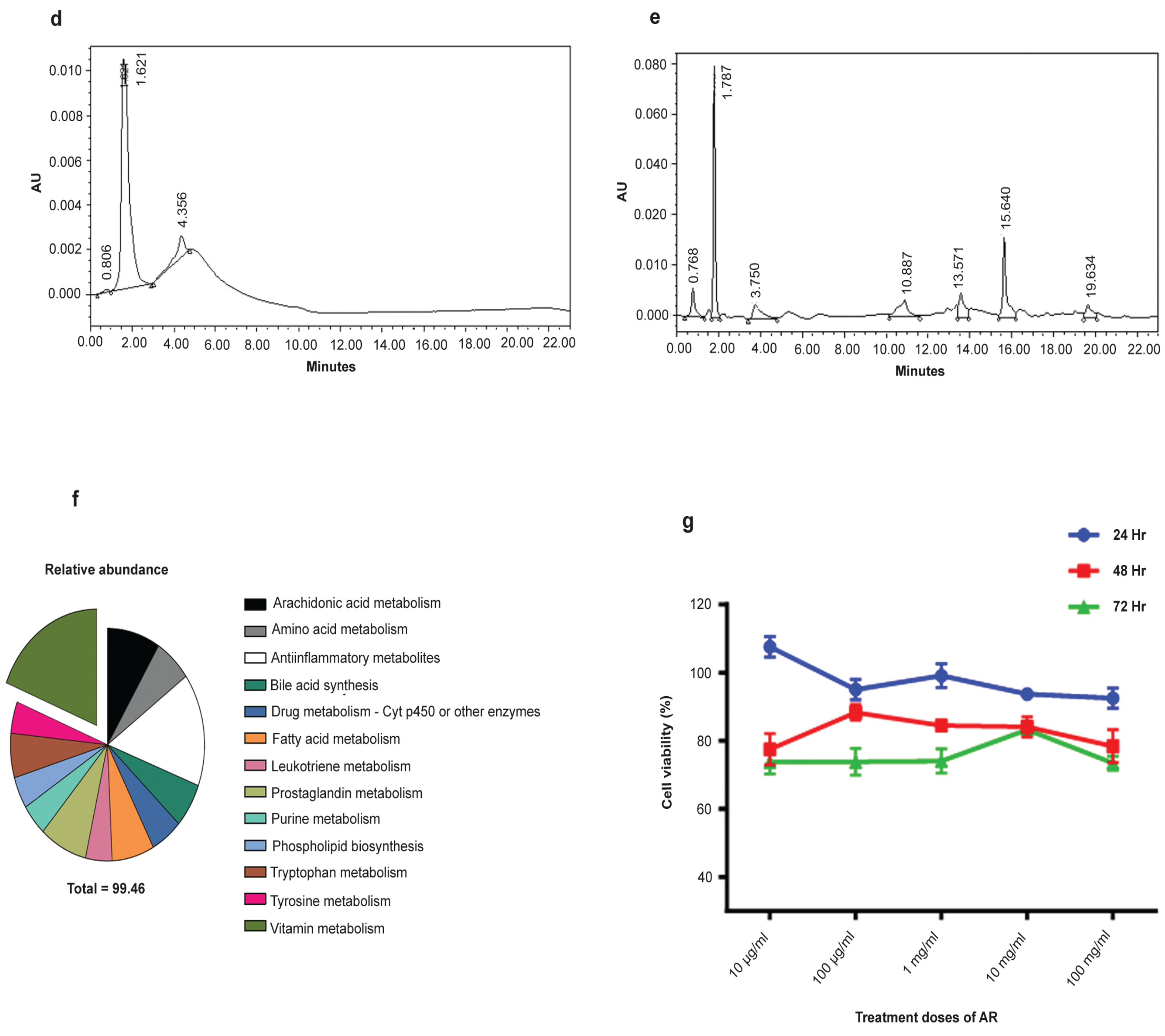

Supplementary Fig. S2

Outline of experimental design

a

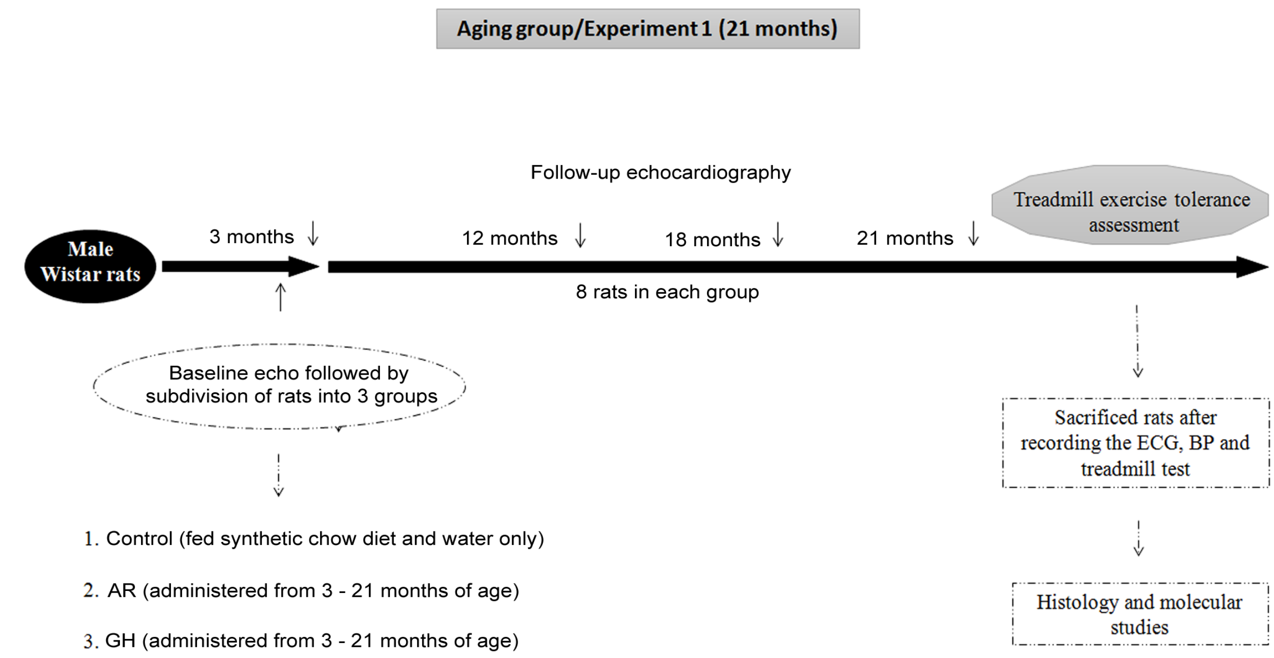

b

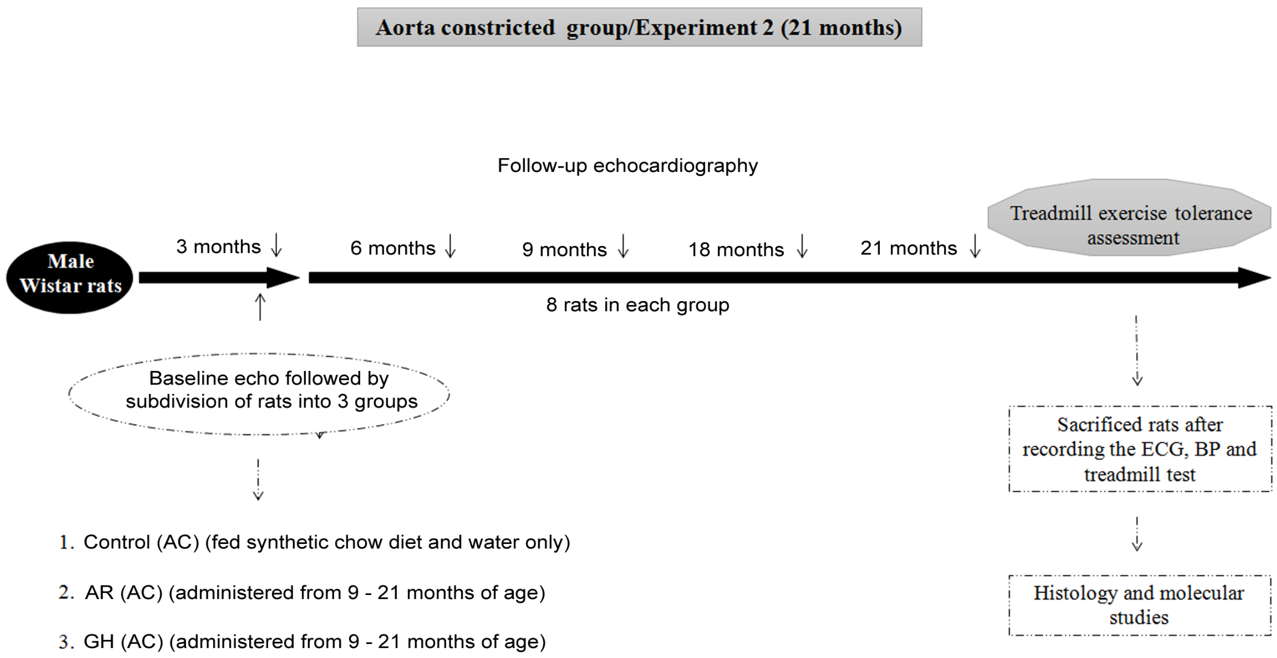

List of supplementary tables.

Supplementary Table S1A. Solubility analysis revealed good solubility of AR in acetonitrile and ethanol while moderate solubility in diethyl ether and methanol. GH had shown good solubility in diethyl ether while moderate solubility in other solvents.

Solvent	Amalaki rasayana/AR	GH
Acetonitrile	+++	++
Ethanol	+++	++
Diethyl ether	++	+++
Methanol	++	++

(+++ Good solubility, ++ Moderate solubility)

Supplementary Table S1B. Details of solvent A and solvent B proportions used in HPLC analysis

Time (min.)	Solvent A (Water : Acetic acid)	Solvent B (Acetonitrile)
0	88	12
3	88	12
8	70	30
13	45	55
15	40	60
17	10	90
21	10	90
23	88	12

Supplementary Table S1C. Retention time from HPLC analysis revealed some common and unique peaks in AR when compared with GH.

Peak	GH		Amalaki rasayana	
	**Ethanol**	**Acetonitrile**	**Ethanol**	**Acetonitrile**
Peak 1	1.621	0.768	0.897	2.155
Peak 2	4.356	1.787	2.030	2.568
Peak 3	-	3.750	4.900	5.482
Peak 4	-	10.887	8.109	7.985
Peak 5	-	13.571	9.465	18.026
Peak 6	-	15.640	-	18.210
Peak 7	-	19.634	-	-

Supplementary Table S2A. Quantitative information of marker compound obtained from AR by HPTLC analysis

S.No.	Sample	Gallic acid (%w/w)	Ellagic acid (%w/w)
1	Raw Amala	0.30	0.09
2	Dried Amala powder	0.90	0.40
3	Amalaki Rasayana (AR)	1.5	0.40

Supplementary Table S2B. Quantitative information of unidentified new peaks (appearing during AR preparation) obtained from AR by HPTLC analysis

**S.No.**	**Sample**	**Marker peak area (AU)**		**Unidentified peaks**		**New peaks**	
		Gallic acid	Ellagic acid	No.	Total area (AU)	No.	Total area (AU)
1	Raw Amala	655.53	331.15	6	1331.13	Nil	Nil
2	Amala dry powder	683.96	214.20	6	571.13	Nil	Nil
3	Amalaki Rasayana (AR)	675.53	320.12	4	696.24	3	315.86

Supplementary Table S3. Comparisons of echocardiography parameters measured at repeated time intervals in aging group of rats.

	**Control (N=8)**			**AR (N=8)**			**GH (N=8)**		
Parameter	3 months (mean± s.d.)	12 months (mean± s.d.)	21 months (mean± s.d.)	3 months (mean± s.d.)	12 months (mean± s.d.)	21 months (mean± s.d.)	3 months (mean± s.d.)	12 months (mean± s.d.)	21 months (mean± s.d.)
IVSd (cm)	0.095± 0.011	0.124± 0.008	0.121± 0.014	0.115± 0.008	0.124± 0.009	0.122± 0.007	0.091± 0.006	0.128± 0.010	0.123± 0.003
IVSs (cm)	0.238± 0.006	0.264± 0.018	0.220± 0.021	0.221± 0.024	0.234± 0.021	0.225± 0.013	0.208± 0.018	0.225± 0.013	0.234± 0.021
LVIDd (cm)	0.632± 0.056	0.661± 0.034	0.653± 0.055	0.681± 0.066	0.716± 0.058	0.752± 0.085	0.698± 0.092	0.731± 0.061	0.724± 0.038
LVIDs (cm)	0.360± 0.050	0.449± 0.043	0.446± 0.046	0.373± 0.052	0.422± 0.084	0.389± 0.059	0.419± 0.074	0.504± 0.033	0.489± 0.019
LVPWd (cm)	0.091± 0.001	0.119± 0.012	0.104± 0.003	0.114± 0.008	0.130± 0.013	0.125± 0.008	0.111± 0.004	0.125± 0.007	0.123± 0.003
LVPWs (cm)	0.201± 0.004	0.223± 0.023	0.210± 0.004	0.205± 0.025	0.236± 0.025	0.229± 0.026	0.213± 0.013	0.251± 0.017	0.256± 0.017
LVFS (%)	43.12± 6.74	32.17± 5.65	32.57± 6.21	45.20± 4.26	41.10± 4.57	48.23± 8.90*	39.87± 7.26	31.02± 8.29	32.46± 2.96
LVEF (%)	81.59± 6.70	68.79± 7.77	69.34± 8.52	83.54± 3.92	79.56± 11.07	86.84± 6.60*	85.15± 6.85	67.19± 10.78	69.20± 4.04

Note: * indicates $P<0.05$; AR (3 - 21 months) versus GH / control (3 – 21 months), 2way ANOVA followed by multiple comparisons by Tukey's test.

Supplementary Table S4. Comparisons of echocardiography parameters measured at repeated time intervals in AC group of rats.

	**Control (AC, N=8)**			**AR (AC, N=8)**			**GH (AC, N=8)**		
Parameter	3 months (mean± s.d.)	9 months (mean±s.d.)	21 months (mean± s.d.)	3 months (mean± s.d.)	9 months (mean± s.d.)	21 months (mean± s.d.)	3 months (mean± s.d.)	9 months (mean± s.d.)	21 months (mean± s.d.)
IVSd (cm)	0.085± 0.006	0.141± 0.01***	0.146± 0.01	0.096± 0.01	0.173± 0.03***	0.138± 0.01*	0.089± 0.01	0.149± 0.02***	0.132± 0.01
IVSs (cm)	0.187± 0.018	0.283± 0.01	0.329± 0.01	0.191± 0.02	0.291± 0.04	0.283± 0.02	0.164± 0.01	0.329± 0.03	0.351± 0.02
LVIDd (cm)	0.691± 0.110	0.520± 0.07***	0.546± 0.05	0.594± 0.05	0.528± 0.03***	0.641± 0.07	0.597± 0.07	0.510± 0.06***	0.509± 0.05
LVIDs (cm)	0.267± 0.032	0.348± 0.06***	0.381± 0.05	0.310± 0.04	0.356± 0.04***	0.427± 0.06	0.346± 0.06	0.359± 0.08	0.372± 0.03
LVPWd (cm)	0.083± 0.005	0.138± 0.01***	0.147± 0.01	0.102± 0.01	0.165± 0.03***	0.136± 0.01*	0.090± 0.01	0.152± 0.02***	0.144± 0.01
LVPWs (cm)	0.177± 0.010	0.296± 0.02	0.279± 0.02	0.190± 0.01	0.261± 0.04	0.261± 0.03	0.177± 0.01	0.241± 0.06	0.263± 0.04
LVFS (%)	61.03± 3.88	32.88± 7.46	30.42± 4.85	47.95± 4.43	32.66± 6.39	33.57± 5.49	42.37± 3.42	29.41± 9.47	26.49± 6.41
LVEF (%)	93.92± 1.84	68.80± 9.66	65.88± 6.89	85.64± 3.37	68.73± 9.11	70.17± 6.97	80.68± 3.39	63.62± 13.89	59.47± 10.77

Note: * indicates $P<0.05$ and *** indicates $P<0.001$; AR (AC, 9 - 21 months) versus GH / control (AC, 9 – 21 months), 2way ANOVA followed by multiple comparisons by Tukey's test.

Supplementary Table S5. Quantification of trichrome stained heart sections of rats from aging and AC group of rats sacrificed at 21 months of age.

Treatment groups				% change in D_{maj}		% change in D_{min}	
	D_{maj} (µm)	D_{min} (µm)	D_{maj}/ D_{min}	AR versus Control	AR versus GH	AR versus Control	AR versus GH
Control	82.25±2.52	19.90±1.50	4.13±0.19	---	---	---	---
AR 250	82.01±3.55	19.01±4.89	4.31±0.01	0.29	1.60	4.47	5.38
AR 500	80.01±1.91	17.02±1.98	4.70±0.24	2.72	3.66	14.47*	14.94*
AR 750	81.02±5.99	18.14±3.99	4.47±0.11	1.50	3.71	8.84*	10.99*
GH 250	83.34±8.70	20.09±1.52	4.15±0.28	---	---	---	---
GH 500	83.05±5.59	20.01±1.95	4.14±0.78	---	---	---	---
GH 750	84.14±6.62	20.38±2.90	4.13±0.17	---	---	---	---
Control (AC)	86.29±7.25	23.09±1.57	3.74±0.08	---	---	---	---
AR (AC)	81.99±6.93	19.10±2.27	4.29±0.16	4.98*	4.83*	17.28*	16.88*
GH (AC)	86.15±8.05	22.98±1.67	3.75±0.17	---	---	---	---

Note: * indicates $P<0.05$; AR versus GH / control and AR (AC) versus GH (AC)/ Control (AC) groups; analyzed by 2way ANOVA followed by multiple comparisons by Tukey's test.

Supplementary Table S6. NOAEL (No Observed Adverse Effect Level) of AR administered rats sacrificed at 21 months of age.

Treatment groups	Heart (n=8) (Mean±SD)		Lung (n=8) (Mean±SD)		Liver (n=8) (Mean±SD)		Kidney (n=8) (Mean±SD)		Body weight(n=8) (Mean±SD)
	Absolute weight (g)	Relative weight (HW/BW, mg/g)	Absolute weight (g)	Relative weight (Lung W/BW, mg/g))	Absolute weight (g)	Relative weight (Liver W/BW, mg/g)	Absolute weight (g)	Relative weight (Kidney W/BW, mg/g)	Absolute weight (g)
Control	1.43± 0.44	4.17± 1.37#	3.67± 1.32	10.70± 4.33	8.22± 1.14	23.96± 5.04	1.43± 0.21	4.17± 0.64	343± 35.97
AR 250	1.63± 0.26	3.62± 0.71	4.28± 0.61	9.50± 1.95	12.41± 1.70	27.53± 1.31	1.87± 0.26	4.15± 0.50	450.67± 46.59
AR 500	1.44± 0.10	3.15± 0.46#	4.66± 0.55	10.18± 1.07	13.31± 1.33	29.06± 3.36	1.75± 0.20	3.83± 0.52	458± 16.82
AR 750	1.45± 0.22	3.38± 0.40	4.18± 0.42	10.08± 1.57	11.86± 0.75	28.59± 3.02	1.61± 0.05	3.88± 0.25	414.77± 35.21
GH 250	2.14± 0.22	4.91± 1.61	4.63± 1.53	10.64± 3.62	14.32± 2.32	32.90± 4.15	1.89± 0.37	4.34± 0.90	435.2± 22.58
GH 500	2.15± 0.51	4.47± 1.00#	4.53± 1.43	9.42± 2.84	12.72± 3.11	26.46± 6.13	1.71± 0.45	3.56± 0.89	480.8± 18.28
GH 750	2.04± 0.26	5.02± 0.69	4.02± 0.14	9.90± 0.69	12.99± 1.08	32.00± 2.31	1.89± 0.31	4.66± 0.79	406± 36.47

Note: # indicates p=0.124; AR-500 versus control group and p=0.176; AR-500 versus GH-500 group; analyzed by 2way ANOVA followed by multiple comparisons by Tukey's test.

Supplementary Table S7. Comparison of function of AR with Western medicines.

S. No.	Compound	Function
1.	Amalaki rasayana (AR)	Improve cardiac function, exercise tolerance, muscle contractility<br>Increase mitochondrial bioenergetics (fatty acid metabolism)<br>Activate adrenergic receptors / Sympathomimetic activity<br>Increase autophagy and level of antioxidant enzymes in aged rats
2.	Digoxin[1]	Improve cardiac function (Increase ejection fraction, cardiac output)<br>Sympathomimetic activity
3.	Nor-adrenaline[2]	Activate adrenergic receptor and downstream signaling<br>Improve cardiac function and muscle contraction<br>Increase fatty acid metabolism
4.	Carnitine[3]	Increase fatty acid metabolism and exercise tolerance in aged rats
5.	CoQ[4]	Antioxidant<br>Increase myocardial bioenergetics<br>Improve exercise tolerance in aged rats

Note: [1]Reference 49, [2]Reference 17, [3]Reference 50, [4]Reference 51 in the manuscript.

Supplementary table S8. Composition of Ghee used in AR.

Sr. No.	Composition of Ghee	Quantity (per ml)
1.	Energy	8 Calorie
2.	Total fat	1 g
3.	Saturated fat	1 g
4.	Polyunsaturated	0 g
5.	Monounsaturated	0 g
6.	Trans	0 g
7.	Cholesterol	2 mg
8.	Sodium	0 mg
9.	Potassium	0 mg
10.	Total carbohydrates	0 g
11.	Dietary fibers	0 g
12.	Sugars	0 g
13.	Protein	0 g
14.	Vitamin A	0 %
15.	Vitamin C	0 %
16.	Calcium	0 %
17.	Iron	0 %

Supplementary Table S9. Details of primers used in qRT-PCR experiments.

Serial #	Gene	Primer Sequence
**1**	**GAPDH**	Forward primer: 5’-CCATGTTTGTGATGGGTGTG-3’<br>Reverse primer: 5’-ACAGTCTTCTGAGTGGCAGTGA-3’
**2**	**BNP**	Forward primer: 5’-AACAATCCACGATGCAGAAG-3’<br>Reverse primer: 5’-GTGCCATCTTGGAATTTCG-3’
**3**	**SERCA2**	Forward primer: 5’-TTCGAAGTCTGCCTTCTGTG-3’<br>Reverse primer: 5’-CTCCAATGGGTGCATAGGTT-3’
**4**	**Atp1a1**	Forward primer: 5’-GAAAGGGACATGGACGAACT-3’<br>Reverse primer: 5’-CCGACAGAATTTGACCCACT-3’
**5**	**TERT**	Forward primer: 5’-CCAGCGTGCATAGCTACTTG-3’<br>Reverse primer: 5’-TGGCACAAATTTGGTACAGG-3’
**6**	**TERF1**	Forward primer: 5’-TGTAGCTGTGTGTATGGAAATGG-3’<br>Reverse primer: 5’-AATTTTCTTTCTAAAGGCGTGTAA-3’
**7**	**COL1a2**	Forward primer: 5’- TTGACCCTAACCAAGGATGC-3’<br>Reverse primer: 5’- CACCCCTTCTGCGTTGTATT-3’
**8**	**PLN**	Forward primer: **5’-**ACCTTACTCGCTCGGCTATC-3’<br>Reverse primer: 5’-GCAGATCAGCAGCAGACATA-3’
**9**	**CAT**	Forward primer: 5’-GAGAGCGGATTCCTGAGAGA-3’<br>Reverse primer: 5’-GAGGGTCACGAACTGTGTCA-3’
**10**	**GPX1**	Forward primer: 5’-CACCGAAATGAATGATCTGC -3’<br>Reverse primer: 5’-GAGCCTTCTCACCATTCACC -3’

**11**	**SOD2**	Forward primer: 5'-AACCCAAAGGAGAGTTGCTG-3'<br>Reverse primer: 5'-ATAAGGCCTGTGGTTCCTTG-3'
**12**	**SOD1**	Forward primer: 5'-ACACAAGGCTGTACCACTGC-3'<br>Reverse primer: 5'-CACCATAGTACGGCCAATGA-3'
**13**	**p53**	Forward primer: 5'-GTTCCGAGAGCTGAATGAGG-3'<br>Reverse primer: 5'-GCCCCACTTTCTTGATCATT-3'
**14**	**ADRB1**	Forward primer: 5'-ACGCTCACCAACCTCTTCAT-3'<br>Reverse primer: 5'-ACATAGCACGTCTACCGAAGC-3'
**15**	**ADRB2**	Forward primer: 5'-TTGGCGTGTGCTGATCTAGT-3'<br>Reverse primer: 5'-TAGCGATCCACTGCAATCAC-3'
**16**	**CACNA1S**	Forward primer: 5'-TCCTCAACTCCATCTTCAAGG-3'<br>Reverse primer: 5'-ACGATGTCTGTCCCAATGAA-3'

Supplementary table S10. Details of antibodies used in Western blot experiments.

Rabbit monoclonal anti- β Tubulin	Cell signaling
Mouse monoclonal anti-SERCA2	Cell signaling
Goat polyclonal anti-BNP	Santa Cruz
Mouse monoclonal anti-cardiac Troponin T	Abcam
Goat polyclonal anti – CREB-1	Santa Cruz
Rabbit polyclonal anti – pCREB-1	Santa Cruz
Rabbit polyclonal anti – pAMPK	Cell signaling
Rabbit polyclonal anti – AMPK	Cell signaling
Rabbit polyclonal anti – Beclin-1	Santa Cruz
Rabbit polyclonal anti – p62	Santa Cruz
Rabbit polyclonal anti – LC3	Santa Cruz
Mouse monoclonal anti- p53	Abcam
Mouse monoclonal anti- ATP5a1	Cell signaling
Rabbit polyclonal anti – COXIV	Cell signaling
Rabbit polyclonal anti – SDHA	Cell signaling
Rabbit polyclonal anti – SOD2	Cell signaling
